# Optimization of metal polymer friction pair composition for hydrogen wear reduction through thermal stabilization analysis

**DOI:** 10.1038/s41598-025-86738-w

**Published:** 2025-01-25

**Authors:** Dmytro Volchenko, Ivan Kernytskyy, Yuriy Royko, Mykola Ostashuk, Nataliia Fidrovska, Vasyl Skrypnyk, Dmytro Zhuravlev, Nataliia Klochko, Vasyl Rys, Oksana Berezovetska, Saurav Dixit, Anna Stefańska, Eugeniusz Koda, Subhav Singh, Kamal Sharma, Rajesh Mahadeva

**Affiliations:** 1 Ivano-Frankivsk National Technical University of Oil and Gas, Ivano-Frankivsk, Ukraine; 2 Institute of Civil Engineering, Warsaw University of Life Sciences, Warsaw, Poland; 3 Lviv Polytechnic National University, Lviv, Ukraine; 4 Kharkiv National Automobile and Road University, Kharkiv, Ukraine; 5 Lviv National University of Nature Management, Dubliany, Ukraine; 6 Centre of Research Impact and Outcome, Chitkara University, Rajpura, Punjab 140417 India; 7 Woxsen Business School, Woxsen University Hyderabad, Hyderabad, India; 8 Division of Research & Innovation, Uttaranchal University, Dehradun, India; 9 Division of Research and Development Cell, Lovely Professional University, Phagwara, Punjab India; 10 Chitkara Centre for Research and Development, Chitkara University, Chandigarh, Himachal Pradesh 174103 India; 11 Department of Mechanical Engineering, Institute of Engineering and Technology, GLA University, Mathura, India; 12 Department of CSE, Manipal Institute of Technology, Manipal Academy of Higher Education, Manipal, 576104 Karnataka India

**Keywords:** Hydrogen wear, Brake friction, Electrolyte, Metal-polymer, Thermal stabilization, Engineering, Mathematics and computing

## Abstract

The composition of the metal-polymer friction pair is carefully considered for interacting with water and hydrogen, ensuring the metals electrode process potential remains below waters in a neutral medium. Simultaneously, adherence to defined chemical composition ratios for the metal-polymer materials is crucial. This analysis is conducted under conditions of thermal stabilization, characterized by a minimal temperature gradient across the rim thickness within an equivalent thermal field. Using the quasi-chemical approximation, the paper derives a concentration-dependent diffusion coefficient of hydrogen (H) in iron (Fe) across a broad spectrum. This derivation includes electronic and vibrational contributions to the chemical potential. The research establishes a correlation between the equivalent diffusion coefficient and the concentration of diffusing hydrogen atoms from the metal, such as the pulley or drum rim. These findings offer novel insights into optimizing hydrogen wear behaviour in brake friction couples, contributing to advancements in materials and design considerations in the automotive field.

## Introduction

The escalating wear of components within road construction machines is a complex challenge beyond the physical deterioration of individual parts; it permeates the very essence of these crucial vehicles, impacting their efficiency and safety in profound ways^1–3^. In the United States, research conducted by the American Society of Civil Engineers (ASCE) has revealed that deteriorating road construction machines contribute significantly to project delays and inefficiencies, imposing substantial economic costs^4^. Similarly, in Europe, data from the European Transport Safety Council (ETSC) underscores the safety risks posed by wear-related issues, particularly in countries like Germany and France, where construction-related accidents are prevalent. Meanwhile, in developing regions like Southeast Asia, wear-induced emissions from inefficient machinery exacerbate environmental concerns, contributing to air and noise pollution in urban construction areas^5,6^. Financially, Australia’s construction industry faces significant losses due to increased maintenance costs and downtime caused by wear-related challenges, as highlighted by the Australian Construction Industry Forum (ACIF). Likewise, in Latin America, countries such as Brazil and Mexico grapple with productivity hurdles stemming from machine breakdowns and maintenance demands^7–9^. This challenge is not isolated; instead, it unfolds as a multifaceted issue, setting in motion a series of consequences that intricately interconnect with critical facets of machine functionality, initiating a far-reaching ripple effect on various operational parameters. As wear advances, its repercussions extend across different layers of machine performance, reaching beyond the realm of individual components to affect the collective efficiency of the entire system. The mechanism by which wear affects machine performance involves a cascade of repercussions propagating through various system layers. Initially, wear begins at the surface of individual components due to friction, abrasion, or mechanical stress. As these components degrade over time, their functionality diminishes, leading to inefficiencies within the machine^10,11^. However, the impact of wear is not limited to isolated components; instead, it permeates throughout the entire system. As wear progresses, it can compromise the alignment, balance, and overall integrity of critical machine parts. This can result in increased friction, reduced tolerance levels, and altered mechanical properties, further exacerbating wear and accelerating deterioration.

Wear and degradation in road construction machines result from friction, abrasion, and exposure to harsh environments. Hydrogen-induced wear, caused by water exposure, leads to metal embrittlement, while thermal stress from uneven temperature distributions accelerate fatigue in components like brakes and gears. Wear-induced debris further damages adjacent parts, disrupting system alignment and efficiency. Environmental factors, such as wet conditions, amplify corrosion and wear, emphasising the need for advanced material designs and maintenance strategies to enhance durability and safety in demanding operations.

Water and hydrogen significantly contribute to wear in construction machinery by accelerating corrosion and causing hydrogen embrittlement. Hydrogen atoms from water diffuse into metal surfaces, weakening the material and exacerbating wear, particularly in high-stress components like brakes and gears. In wet conditions, water also promotes debris formation, leading to secondary damage and further compromising machinery integrity. Addressing these effects is crucial for improving durability in demanding environments.

Additionally, wear-induced debris and contaminants may circulate within the machine, causing secondary damage to adjacent components and subsystems. Furthermore, the cumulative effects of wear can disrupt the harmonious interaction between different components, leading to decreased coordination, synchronization, and overall system performance. This can manifest as reduced operational efficiency, increased energy consumption, and compromised output quality. Ultimately, the repercussions of wear extend beyond the realm of individual components to affect the collective efficiency and functionality of the entire machine system^12^.

The multifaceted nature of this challenge arises from the intricate relationships between the diverse components constituting road construction equipment and their crucial roles in the overall operation of these machines. From an efficiency standpoint, wear-induced degradation creates a domino effect on the machine’s ability to perform optimally. It influences the specific parts experiencing wear and crucial aspects such as engine power, traction qualities, and braking efficiency^13,14^. This creates a cumulative decline in operational effectiveness, as the wear and tear in these components synergistically compromise the machine’s overall performance. Moreover, the safety implications are profound and extend well beyond operational inefficiencies. Wear-related challenges pose inherent risks to the personnel operating the machines and the environment in which they operate. Dealing critical components, particularly braking systems, introduces safety hazards that can result in accidents and injuries. This emphasizes the need for a holistic approach to address wear-related issues, incorporating efficiency considerations and a robust focus on safety standards and risk mitigation strategies^15,16^.

### Engine power and traction

The impact of wear on engine power and traction qualities constitutes a critical aspect of the multifaceted challenges faced by road construction machines. As components within these machines experience wear, there is a consequential decline in engine power. This reduction in engine power goes beyond being a localized issue; it permeates the overall performance of the machines. The reduction in engine power has direct implications for the ability of road construction machines to operate optimally. These machines are often tasked with handling heavy loads and navigating challenging terrains on construction sites. The compromised engine power hampers their capacity to efficiently execute these tasks, resulting in a slowdown of construction operations.

Furthermore, the decline in traction qualities is a significant repercussion of wear-induced engine power reduction^17,18^. Traction is paramount for road construction machines, as it determines their ability to grip and move across various surfaces. Diminished traction can impede the machines’ ability to navigate and operate effectively, particularly when adverse or uneven terrain conditions. The combined effect of reduced engine power and compromised traction introduces operational inefficiencies beyond mere performance degradation. It can result in delays in construction projects, increased fuel consumption as machines struggle to perform, and heightened maintenance requirements due to the additional strain on the machinery.

### Braking efficiency

The wear-induced impact on the braking system is a pivotal concern in the broader challenges road construction machines face. The braking system, a critical component ensuring operational safety, undergoes wear in its metal friction elements, contributing to a noticeable decrease in braking efficiency. Brakes play a paramount role in safely operating vehicles and handling equipment on construction sites^19,20^. As these machines often handle substantial loads and operate in dynamic environments, the reliability of the braking system is essential for ensuring the safety of both machine operators and the surrounding environment. The wear in the metal friction elements of brakes introduces a progressive degradation in braking efficiency over time. This deterioration is not merely a matter of performance; it directly correlates with the ability of road construction machines to come to a controlled stop, particularly when faced with challenging or unexpected situations on construction sites.

**Fig. 1:**
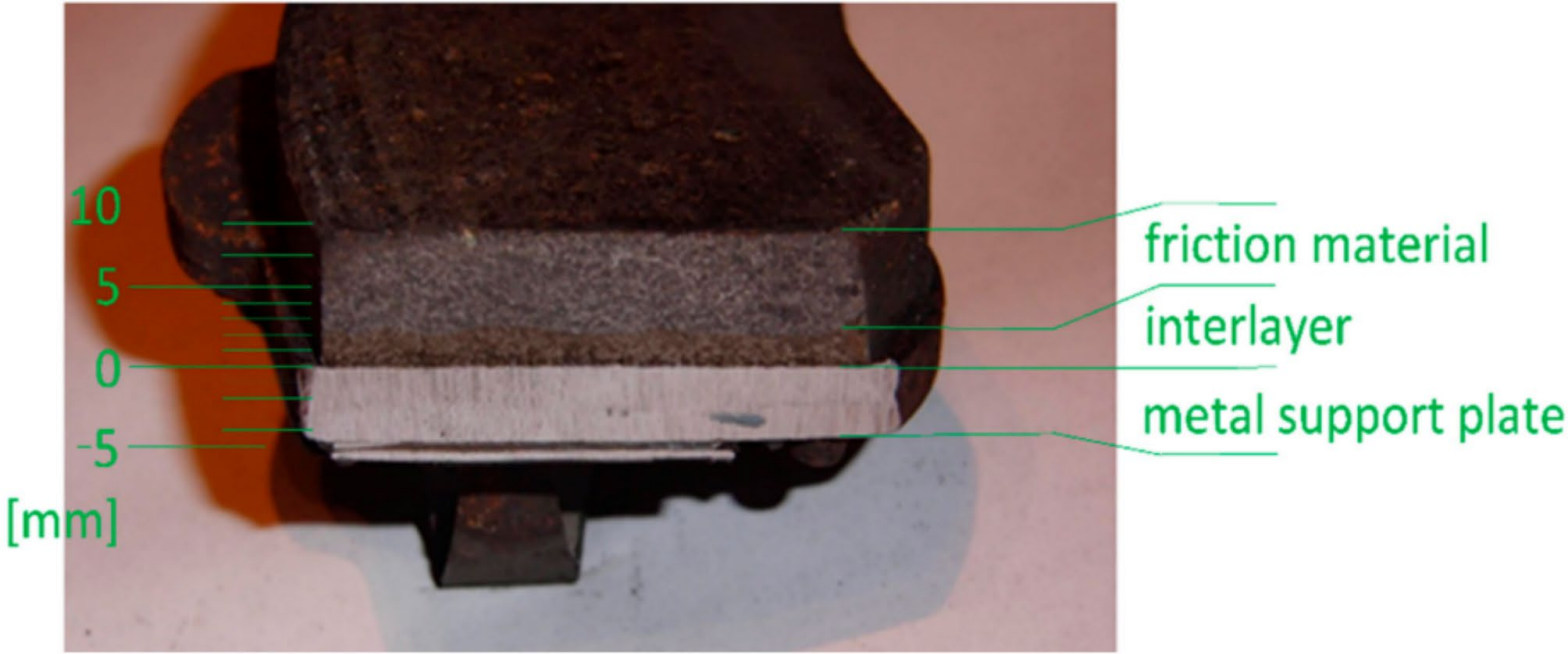
Cross-sectional view showing the three distinct layers in the first group of pads: the metal support plate, the binder layer (interlayer), and the friction material. Adapted from Ref^22^. under CCBY 4.0.

The consequences of reduced braking efficiency are profound and extend beyond the operational aspects of the machines. Safety becomes a primary concern as reliable brakes safeguard against accidents, collisions, and unintended movements. In environments where precision control is crucial, such as construction sites with heavy equipment and personnel, compromised braking efficiency poses significant risks^21^. Figure 1 shows the initial set of pads, wherein it has been shown that the three distinct layers, the metal support plate, an approximately 3 mm thick binder layer (interlayer), and the friction material, are crucial for the effective braking system.

Addressing wear in the braking system is imperative for maintaining high safety in road construction operations. Regular inspections, timely maintenance, and the incorporation of advanced braking technologies become crucial strategies in mitigating the wear-induced decline in braking efficiency^23,24^. By ensuring the reliability of brakes, road construction machines can operate with enhanced safety measures, protecting both operators and the integrity of the construction site.

### Goods safety

The wear-induced challenges in road construction machines extend beyond their immediate operational implications and significantly impact the safety of transported goods. As various machine parts experience wear and operate below optimal conditions, a chain reaction of risks emerges, posing potential hazards to the goods being transported. Machines operating below optimal conditions due to wear are susceptible to malfunctions and failures. Transporting goods introduces a high degree of uncertainty and risk. Malfunctions may lead to unexpected stops or breakdowns during transit, resulting in delays and disruptions to the smooth flow of goods through the supply chain. Accidents become a heightened risk when machine parts, compromised by wear, fail to function as intended^25,26^. A failure in critical components can lead to the loss of control over the machinery, potentially resulting in collisions or accidents that jeopardize the safety of the transported goods and pose a threat to personnel in the vicinity.

The potential for damage to goods directly results from wear-induced challenges in road construction machines. The machinery’s inability to operate optimally increases the likelihood of rough handling, sudden stops, or uncontrolled movements, all of which can damage goods during transport. Disruptions to the supply chain represent another significant consequence of wear-related challenges. Unplanned stops or delays in the transportation process can have cascading effects on the overall logistics and scheduling of goods delivery. This can result in financial losses, strained customer relationships, and a compromised reputation for reliability in the supply chain. Addressing wear-related challenges in the context of goods safety requires a proactive approach^27^. Regular maintenance, thorough inspections, and timely replacement of worn components are essential to mitigate the risks associated with wear-induced machinery malfunctions. Ensuring the reliability and optimal performance of road construction machines is crucial for operational efficiency and safeguarding the safety and integrity of the goods being transported.

### Safety of personnel and passengers

The challenges posed by wear-related issues in road construction machines directly and significantly impact the safety of both the personnel operating these machines and any passengers on board. The reliability of machine performance, especially during critical functions such as braking and manoeuvring, becomes a paramount concern as wear progresses^28,29^. Unreliable machine performance due to wear increases the risk of accidents and injuries. This unreliability can manifest in various ways, including decreased engine power, compromised traction, and reduced braking efficiency, all of which are critical for ensuring the safe operation of road construction machines. During braking, the wear-induced decline in braking efficiency can result in longer stopping distances and diminished control over the machines. This poses a direct threat to the safety of operators and passengers, especially in situations requiring sudden stops or precise control, which is common in the dynamic environments of construction sites.

Manoeuvring, which involves the coordinated movement of the machine, becomes precarious as wear impacts critical components. Unreliable manoeuvring increases the likelihood of accidents, collisions, or unintended movements, putting operators and passengers at risk of injury^30,31^. The potential consequences of wear-related challenges extend beyond physical injuries. Accidents or malfunctions due to wear can lead to psychological stress for the personnel operating the machines and erode the overall confidence in the safety of the working environment.

### Water-containing environment

The presence of water in the operational environment of road construction machines introduces a distinct set of challenges, particularly exacerbating wear-related issues. This environmental factor, when coupled with the friction and mechanical stresses inherent in the operation of these machines, contributes significantly to hydrogen wear on the metal surfaces of crucial components. Water-induced hydrogen wear is a phenomenon where the interaction between water and metal surfaces leads to the generation of hydrogen atoms^32,33^. These hydrogen atoms can diffuse into the metal, causing structural changes and accelerating wear. This is particularly relevant in road construction machines, where the equipment is frequently exposed to wet or damp conditions. The accelerated degradation of crucial components due to water-induced hydrogen wear has profound implications for the reliability and longevity of road construction machines. Components such as brake systems, gears, and other metal parts are susceptible to accelerated wear, compromising their performance and overall integrity.

Mitigating the effects of water-induced hydrogen wear necessitates the implementation of effective strategies. Protective measures such as corrosion-resistant coatings, improved seals, and materials less prone to hydrogen embrittlement can be crucial in reducing the impact of water exposure on metal components. Additionally, regular maintenance practices that include inspections for water-related damage and the timely replacement of worn parts become essential for sustaining the optimal performance of road construction machines in such environments. Understanding the interplay between water, metal surfaces, and the resulting hydrogen wear is key to developing targeted and effective mitigation strategies. By addressing these challenges, road construction machinery can be better equipped to withstand the harsh conditions of water-containing environments, ensuring prolonged operational efficiency and minimizing the risk of premature component failure. Mukhametishina et al.^34^ research addressed these challenges by specifically focusing on hydrogen wear in road-building machines. By identifying general patterns and characteristics of hydrogen wear as a unique form of surface destruction, the study sheds light on the intricate mechanisms at play. Understanding how surfaces of road-building machine components undergo hydrogenation provides insights into the root causes of wear. Moreover, formulating methods to protect against tribo-hydrogenation presents practical approaches to mitigate the detrimental effects of hydrogen wear. Additionally, several reports^35–38^ consider the interaction of hydrogen with metals and non-metallic elements. The research illustrated the effect of hydrogen on various properties of metals and alloys and the occurrence of specific defects in them^39^. Diverse information about hydrogen embrittlement and the impact of hydrogen on the mechanical characteristics of the *hydrogen-metal*pair in the groups of D. Mendeleev’s periodic system has been expanded. The work^40^ is devoted to the wear of sub-roughness of friction surfaces in a hydrogen-containing medium. In the research, hydrogen is pumped into the subsurface layer of a metal body and interacts with its crystal lattice. It is noted that the driving forces in the processes of hydrogen wear are the bulk temperature, internal pressure, deformation, structure and defects of the crystal lattice.

Liu and Yudin studied the physical and mechanical processes of the friction surface of hydrogen wear on machine parts and equipment^41,42^. The causes of hydrogen release, hydrogenation of rubbing surfaces and their destruction are established. A complex picture shows the behaviour of hydrogen in surface layers during friction under various factors, and the impact of “biographical” hydrogen on the wear of parts is determined. The reasons for the transfer during friction of a rigid material to a softer material are stated: steel to bronze, cast iron to plastic. Practical recommendations are given to suppress hydrogen wear and increase the durability and reliability of friction units of machines and equipment. At the same time, the following was not considered: the effect of external hydrogen on the surface layer of a metal friction element and its entry into the subsurface layer by injection; the phenomenon of adhesion and the types of contacts of friction pairs during their frictional interaction, as well as the combination of adsorption-diffusion phenomena observed in the surface and subsurface layers of friction pairs, were not taken into account.

Most importantly, external and internal hydrogen and their role in tribological reactions have not been isolated. In a report^34,37^, the maximum surface-volume temperature is formed at a certain depth from the friction surface under severe friction conditions. This creates conditions under which hydrogen, if it is adsorbed on the surface of the part, diffuses deep into the surface under a temperature gradient, concentrates there, causes embrittlement of the surface layers, and increases wear. However, what happens in the subsurface layer of a metallic element with the structures of its crystal lattices was not indicated. Smirnov^43^ devoted to diffusion and behaviour patterns of the hydrogen subsystem in the *metal-hydrogen* systems. The latter deals with one-component metals and two-component alloys. Hydrogen elasticity was introduced to hydrogen for the first time.

The primary objective of studying the composition of metal-polymer friction pairs in the context of hydrogen wear reduction is to optimise material interactions to minimise wear and enhance performance in hydrogen-exposed environments. This involves ensuring that the electrode process potential of the metal component remains lower than that of water in a neutral medium to prevent adverse electrochemical reactions. Additionally, careful regulation of the chemical composition ratios of the metal and polymer components is essential to achieve optimal durability and thermal stabilisation. These considerations aim to mitigate hydrogen-induced wear mechanisms, which compromise material integrity, particularly in applications such as braking systems. By providing a stable thermal field, thermal stabilisation enables a precise correlation between the equivalent diffusion coefficient of hydrogen and its concentration, offering insights into the mechanisms of hydrogen-induced embrittlement and wear. This approach facilitates accurate modelling of hydrogen behaviour within the metal matrix, which is critical for developing strategies to reduce wear and enhance the durability of metal components in demanding applications, such as braking systems. This approach provides a framework for developing friction pairs with improved longevity and efficiency under challenging operational conditions.

The article’s content encompasses a comprehensive exploration of several key questions about the characteristics of electrolytes and their interaction with metal components. The material delves into the intricate dynamics involved in the interaction of metal components with water and hydrogen, shedding light on the complexities of these relationships. Furthermore, the investigation extends to understanding the behaviour of atoms within the hydrogen-metal system. The article culminates in a detailed discussion of the obtained results, specifically addressing the overarching issue of reducing hydrogen wear in brake friction pairs.

## Characteristics of electrolytes and their interaction with metal components

Polymers commonly used in brake systems, such as epoxy resins, polyurethane, and phenolic resins, play a critical role in determining the performance and durability of friction materials. These polymers are selected for their thermal stability, mechanical strength, and resistance to wear under high-stress conditions. However, their interactions with hydrogen present unique challenges. Hydrogen exposure can lead to polymer degradation, including embrittlement, reduced mechanical integrity, and altered frictional properties. For example, hydrogen diffusion into the polymer matrix may weaken interfacial bonds, compromising the composite material’s overall performance. Among these polymers, phenolic resins exhibit superior thermal resistance, making them less susceptible to hydrogen-induced degradation under high-temperature conditions. Understanding these interactions is essential for improving material formulations and developing brake systems that maintain their structural integrity and efficiency in hydrogen-rich or moisture-prone environments.

Electrolytes, in the context of substance solutions or melts, are crucial components with the unique ability to conduct electricity. This phenomenon occurs due to the presence of ions, which are electrically charged particles formed due to the dissociation of electrolyte molecules within the solution or melt. The key characteristic that distinguishes electrolytes is their capacity to divide or dissociate into these electrically charged ions, and this ability varies for different substances^44,45^. The quantitative measure of this dissociation ability is expressed by the degree of dissociation, denoted as α. This parameter is determined by evaluating the ratio of the number of molecules that have undergone dissociation to the total number of molecules initially dissolved in the solution. In simpler terms, α represents the proportion of electrolyte molecules that have split into ions, indicating the extent to which the substance contributes to the conductivity of the solution. The degree of dissociation, α, is a crucial factor in categorizing electrolytes into two main types: strong and weak. In strong electrolytes, the degree of dissociation is large (α is close to 1), signifying that almost all molecules have separated into ions. Conversely, weak electrolytes exhibit a small degree of dissociation (α is relatively small), indicating that only a fraction of the molecules have dissociated into ions. The mechanism and features of the behaviour of ions describe the Arrhenius and Debye-Hückel theorem. In the study of electrolyte solutions, a fundamental distinction is made between two types of electrical conductivity: specific (ϰ) and equivalent (λ). Specific electrical conductivity, denoted as ϰ, focuses on the conductivity of a defined volume of the electrolyte solution, precisely one cm³ enclosed between two parallel plates spaced 1 cm apart. This measurement allows for the assessment of the conductivity of a constant volume (1 cm³) while permitting variations in the concentration of the dissolved electrolyte. Consequently, specific conductivity provides valuable insights into how the solution’s conductivity changes in response to alterations in electrolyte concentration, offering crucial information for understanding the relationship between concentration and electrical conductivity^46,47^. On the other hand, equivalent electrical conductivity, represented by λ, takes a different approach by evaluating the conductivity of various solution volumes, all containing a constant amount of electrolyte measured in equivalents (1 g-eq). The equivalent, which indicates the amount of electrolyte corresponding to one mole of charges produced during dissociation, ensures that the comparison is based on a consistent quantity of active ions. λ provides information on how the conductivity varies for different solution volumes while maintaining the amount of dissolved electrolyte constant. This type of measurement is beneficial when comparing the conductivity of solutions with the same amount of active ions.

They are related to each other by the equation:

1 $$\lambda = \frac{{1000 \cdot \varkappa}}{C},$$

where *С* – solution concentration, expression in g-eq/l.

**Fig. 2:**
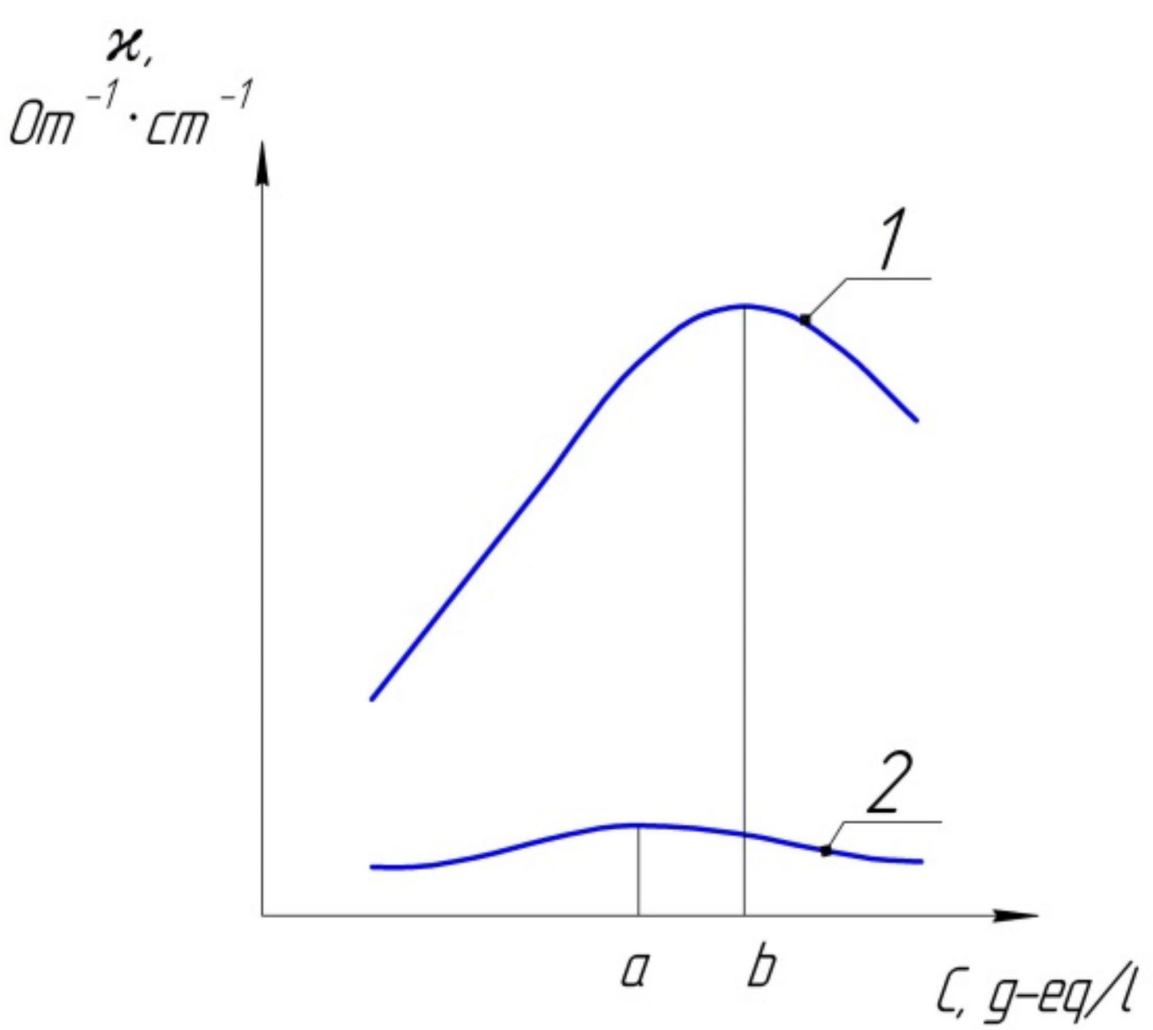
Dependence of electrical conductivity on the concentration of the electrolyte solution: 1 and 2 - strong and weak electrolyte.

**Fig. 3:**
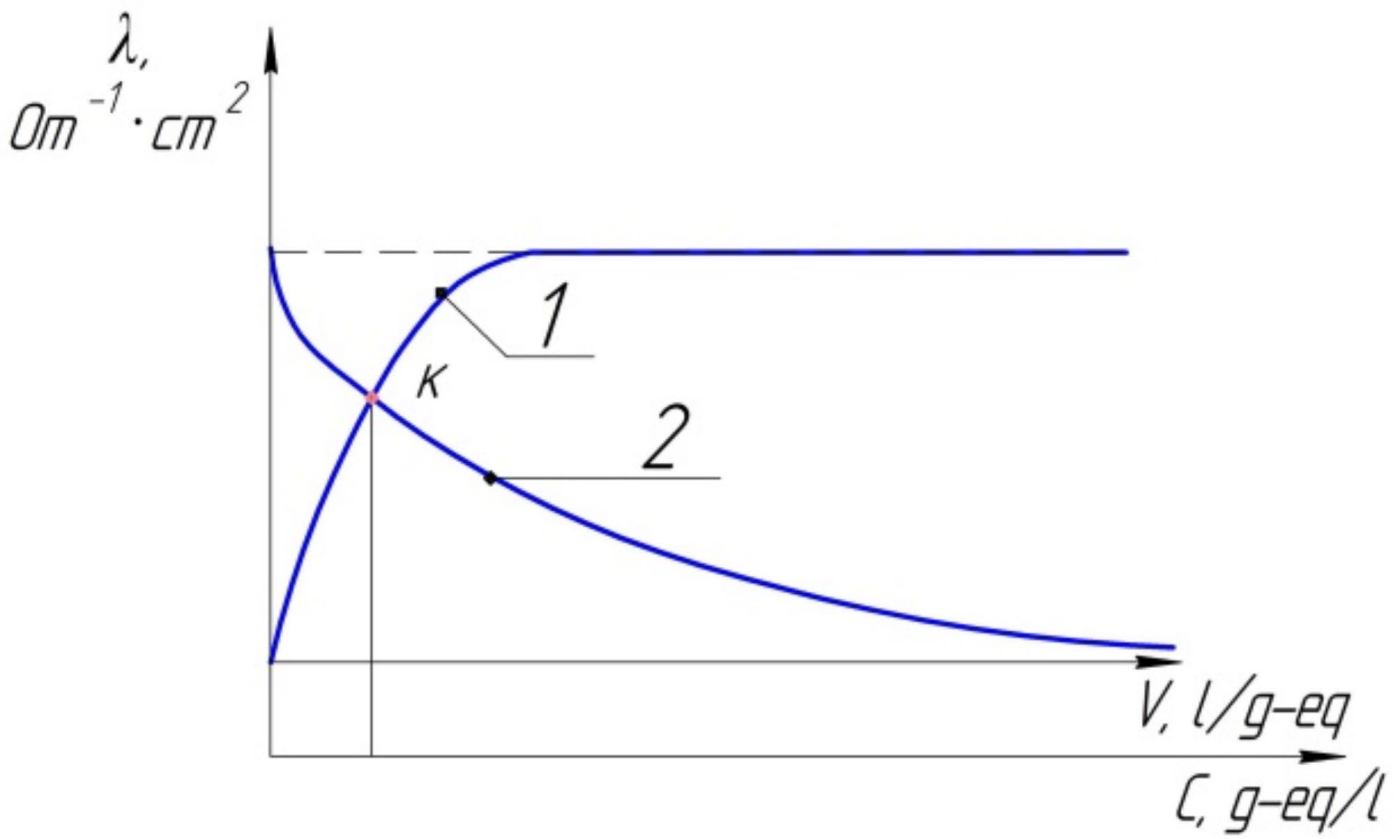
Dependence of the equivalent electrical conductivity on the dilution of the solution (1) and its concentration (2).

The graphical dependencies in Figs. 2 and 3reveal essential patterns related to specific electrical conductivity (ϰ) in electrolyte solutions, particularly concerning concentration variations. In the region of low concentrations, ϰ demonstrates an initial increase with rising concentration. This behaviour is intuitive, as a higher concentration implies more ions in 1 cm³ of the solution, enhancing electrical conductivity. However, as the concentration increases, a critical point is reached where ϰ attains a maximum value before gradually decreasing. This decrease in specific electrical conductivity in solutions of strong electrolytes is attributed to intricate phenomena such as the relaxation effect and electrophoretic braking. The relaxation effect implies a slowing of ion movement, contributing to the observed decline in ϰ^48,49^. Electrophoretic braking, another factor influencing strong electrolytes, further hinders ion movement, reducing electrical conductivity. On the other hand, in solutions of weak electrolytes, the decline in ϰ is associated with a decrease in the degree of dissociation. As concentration increases, the fraction of molecules dissociating into ions decreases, leading to decreased electrical conductivity.

The exploration of the influence of solution concentration on the equivalent electrical conductivity (λ) is conveniently initiated by examining its dependence on the dilution of the solution. Dilution, represented as V = 1/C, where V is the volume and C is the concentration, provides a reciprocal measure of concentration. Through experimental observations, it becomes evident that with increasing dilution (corresponding to decreasing concentration), the equivalent electrical conductivity experiences a consistent increase^50^. This trend is graphically depicted in Fig. 1. The graphical representation reveals a noteworthy observation: the equivalent electrical conductivity reaches a limiting value as dilution progresses. This ultimate value is termed the electrical conductivity at infinite dilution and is denoted as λ∞ (or λ0). The significance of this limiting value lies in its portrayal of the maximum conductivity achievable under the conditions of infinite dilution, where the concentration approaches zero. The point K, identified as the intersection of curves 1 and 2 in Fig. 3, is particularly noteworthy. At this point, both strong and weak electrolytes exhibit the same mobility of ions in the electrolyte, providing a crucial reference point in understanding their behaviour under extreme dilution conditions.

The observed increase in specific electrical conductivity (ϰ) and equivalent electrical conductivity (λ) with rising temperature is rooted in the fundamental changes occurring within electrolyte solutions under thermal influence. This temperature-dependent behaviour can be attributed to several interconnected factors contributing to enhanced ion movement and increased electrical conductivity. Firstly, the rise in temperature leads to a decrease in the viscosity of the medium in which the electrolyte is dissolved. As the viscosity decreases, the resistance to the movement of ions is reduced, facilitating their mobility through the solution^51,52^. This reduction in resistance is a key contributor to the overall increase in electrical conductivity. Secondly, temperature influences the hydration of ions. With an increase in temperature, ions experience partial dehydration, reducing the radius of a hydrated ion. This reduction in hydrated ion size further contributes to a more accessible and rapid movement of ions through the solution, contributing to the heightened electrical conductivity.

Additionally, the increase in temperature is associated with a higher degree of dissociation, particularly noticeable in weak electrolytes. As the temperature rises, weak electrolytes undergo more extensive dissociation, leading to greater ions in the solution. This increase in ion concentration contributes significantly to the overall rise in electrical conductivity. The combined effects of decreased viscosity, partial dehydration of ions, and increased dissociation collectively lead to a substantial improvement in ion mobility, resulting in higher electrical conductivity at elevated temperatures. Understanding these temperature-dependent mechanisms is crucial in various applications, especially in fields such as electrochemistry and materials science, where precise control over electrical conductivity is essential for optimizing the performance of devices and processes.

The interaction of metal components with complex oxidizing agents proceeds depending on the stability of these compounds, which is determined by the temperatures and nature of the medium. The study of the reactions of the interaction of metal components in aqueous solutions is complicated by the hydrolysis of salts and sometimes by the precipitation of hydroxides or basic salts. In aqueous solutions, transforming metal atoms into ions includes several stages: breaking chemical bonds between atoms, ionizing free metal atoms, and hydration of the resulting ions. During the transformation under consideration, energy is expended to break chemical bonds and ionize metal atoms, and energy is released during their hydration. Of the three terms of the total balance, only one energy - the ionization energy - is directly determined by the position of a particular metal in the periodic system. Therefore, the ionization energy cannot unambiguously characterize the activity of metal components in reactions with aqueous solutions. The ionization energy acts only as one of the factors, along with others, that determine the energy effect of the response. A more objective measure of the reduction activity of metal components in aqueous solutions is the electrode potential that occurs at the metal-solution interface^53^.

The electrode potential between the metal and the solution occurs when the metal (Me) is immersed in an aqueous solution of its salt containing hydrated ions [Me(H_2_O)m]n^+^. The reason for the emergence of the electrode potential is the transition of a certain amount from the metal to the solution in the form of positively charged ions as a result of their interaction with water dipoles:

$${\text{Me}}{-}n{\text{e}} + m{\text{H}}_{{\text{2}}} {\text{O}} \rightleftharpoons \left[ {{\text{Me}}\left( {{\text{H}}_{{\text{2}}} {\text{O}}} \right)_{m} } \right]^{{n + }}$$

The metal components acquire a negative charge, and the near-surface layer of the solution enriched with metal ions acquires a positive charge. This leads to the formation of a double electric layer at the “metal-solution” interface and the appearance of a certain potential difference or potential jump^54^. The transition of ions of metal components into solution and back is reversible. If the rates of metal ionization and discharge of its ions are equal, a dynamic equilibrium occurs between the oxidized and reduced forms of the substance. The equilibrium electrode potential is the potential difference at the phase boundary in reversible systems upon the onset of equilibrium between the oxidized and reduced forms of a substance. The equilibrium potential of the metal components is established at the electrode when only ions of this metal participate in the exchange process. The value of a metal’s electrode potential depends on the metal’s nature, the concentration (more precisely, the activity) of the ions of the given metal in solution, and the temperatures.

The value of the equilibrium electrode potential is calculated using general thermodynamic equations. For a metal electrode, the equilibrium potential, which is established when equilibrium is reached, is calculated according to the Nernst equation:

2 $$\:{E}_{\text{M}{\text{e}}^{n+}\text{/Me}}={E}_{\text{M}{\text{e}}^{n+}\text{/Me}}^{O}+\frac{0.059}{n}{lg}\left[\text{M}{\text{e}}^{n+}\right]$$

where ${\text{E}}_{{{\text{Me}}^{{{\text{n}} + }} {\text{/Me}}}}$ - equilibrium electrode potential of the components, V;

${\text{E}}_{{{\text{Me}}^{{{\text{n}} + }} {\text{/Me}}}}^{{\text{O}}}$- standard electrode potential of metal components, V;

*n* – number of electrons donated by metal components, V.

$\left[ {{\text{Me}}^{{n + }} } \right]$ - concentration of ions in metal components, mol/l.

The latter must be comparable values to characterize the properties of metals by the magnitude of their electrode potential. For this purpose, the standard electrode potential of metals are used and measured relative to the potential of a standard hydrogen electrode under the same comparable conditions. The electrode potential of metals measured in a solution of their ions at a temperature of 298 K and the concentration of metal ions in the solution = 1 mol/l are called standard *electrode potentias*^55^.

A series of standard electrode potential is obtained by arranging the metals in ascending order of the algebraic value of their standard electrode potential. The standard electrode potential’s value quantitatively characterizes the metal’s reduction and ions’ oxidizing ability. The lower the algebraic value of the potential, the higher this metal’s reduction ability and the lower its ions’ oxidizing ability. Following the Nernst equation, the redox ability of systems depends on the activity (concentration) of the oxidized and reduced forms of the substance, and for chemical reactions involving H^+^ and OH^-^ ions, it also depends on the pH of the solution.

By the values ​​of the redox potential, it is possible to determine the direction of the spontaneous occurrence of redox reactions. Knowing the value of standard redox potential makes it possible to decide on spontaneous redox reactions’ depth (degree) in a certain direction. The electrode potential measures the change in the Gibbs free energy (ΔG) in the system and thus indicates the direction of the redox process^56,57^. The loss of standard Gibbs energy (ΔGO) is at constant pressure and temperature.

3 $$\Delta {\text{G}}^{O} = - \Delta E^{O} nF$$

где *F* – Faraday number (96485 C);

*n* – number of electrons per transferred ion;

ΔЕ^О^ – difference of standard system potential, V.

Since the sign (-) at ΔG^O^ corresponds to a spontaneous process, the greater the difference ΔE^O^, the more negative the value of the Gibbs energy and the more likely the electrode process. To determine the direction of the spontaneous occurrence of redox processes, it is necessary to calculate the EMF of the system as the potential difference of the redox electrodes involved in this process.

The presented tables offer a comprehensive insight into the chemical composition and interactions of materials crucial to the performance of a metal-polymer friction pair. Table 1 outlines the chemical composition of steel 35KhNL. It is emphasizing its interaction with hydrogen. Understanding how steel components react with hydrogen is pivotal. It influences material properties and durability in relevant applications.

**Table 1:** The chemical composition of the components of the structure of steel 35 KhNL and their interaction with hydrogen.

Chemical elements and their percentage	Interaction with hydrogen
Iron (Fe) (96,6–97,8) *	In the hydrogen-iron system, the last element can have three (*α*-Fe, *γ*-Fe, *δ*-Fe) structural modifications with different lattice types. Iron adsorbs hydrogen, and in any state, the process of hydrogen occlusion by iron has an atomic character. Hydrogen diffuses through the crystal lattice of iron as a proton.
Silicon (Si) (0,20–0,42)	There is no data on metallic silicon’s adsorption and occlusion capacity concerning hydrogen and other gaseous elements.
Copper (Cu) no more than (0,30)	When copper enters the microprotrusions of the rim at a flash point of 1084 °C, copper passes into a liquid state, which leads to a sharp drop in its thermal conductivity^58^. It has been established that the thermal conductivity of liquid copper is two times lower than that of solid metals. There is an intense occlusion between liquid copper and hydrogen.
Manganese (Mn) (0,40–0,90)	At temperatures from 20 °C to 500 °C, hydrogen solubility in manganese decreases; above 500 °C, occlusion becomes endothermic and increases with increasing temperature.
Nickel (Ni) (0,70 − 0,90)	The adsorption and diffusion of hydrogen in nickel have been studied in detail. It seems likely that the hydrogen-containing nickel with a high H/Ni ratio is a metallic bond type composition.
Phosphorus (Р), no more than (0,047)	Phosphorus gives chemical compounds with hydrogen corresponding to PH3 (phosphine) and P2H4 (diphosphine) formulas. The existence of higher phosphorus hydrides has not been revealed.
Chromium (Cr) (0,50–0,80)	Prone to adsorption and desorption at various temperatures. Two new phases are observed in the chromium-hydrogen system. The maximum hydrogen concentration is determined by the H/Cr ratio.
Sulfur (S) no more than (0,04)	At 310 °C, the direct interaction between sulfur and hydrogen reaction proceeds towards the formation of H_2_S. At 400 °C, hydrogen sulfide is already decomposing: its formation at this temperature is possible only when sulfur and hydrogen vapour interact. At 600–650 °C, the content of hydrogen sulfide in the gas mixture is only 7%

*Note: With increasing pressure and temperature, the diffusion rate of hydrogen in iron increases.

The interaction of carbon and hydrogen is discussed below.

A chemical reaction proceeds spontaneously in the forward direction if the EMF of the system is positive:

4 $${\text{EMF}} = \Delta E = E_{{{\text{ox}}}} {-}E_{{{\text{re}}}}> 0.$$

By the value of the electrode potential of the metal components, it is possible to predict the behavior of metals in relation to water, solutions of acids, alkalis, and salts.

## Interaction of metal components with water and hydrogen

Since water contains hydroxonium ions H_3_O^+^, it should act on metals like an acid. However, the concentration of these ions and their lifespan in water is minimal, so the interaction of metals with water is unique^59,60^. In addition, water can be an oxidizing agent with metals and act as a ligand in complex formation processes.

Electrode Process Potential 2Н^+^ + 2е = Н_2_ depends on рН environment and is determined by the ratio ${\text{E}}_{{2{\text{H}}^{ + } /{\text{H}}_{2} }} = {\text{E}}_{{2{\text{H}}^{ + } /{\text{H}}_{2} }}^{{\text{O}}} - 0,059\;{\text{pH}}.$ In a neutral environment at рН = 7, when the oxidizing agent is water, for the reaction

5 $$2{\text{H}}_{2} {\text{O}} + 2{\text{e}} - {\text{H}}_{2} + 2{\text{OH}}$$

The potential of the hydrogen electrode is

6 $${\text{E}}_{{{\text{2H}}_{{\text{2}}} {\text{O/H}}_{{\text{2}}} }} = - 0,059 \cdot 7 = - 0,41\;{\text{B}}.$$

Both conditions must be met for the reaction with metals to proceed with water. In addition, the potential of the metal in a neutral medium must be less than the potential of the oxidizing agent (water):

7 $${\text{E}}_{{{\text{Me}}^{{{\text{n}} + }} {\text{/Me}}}} < {\text{E}}_{{{\text{ox}}}} .$$

The interaction of metals with water is governed by their electrochemical potential and the solubility of the reaction products. Theoretically, only metals with potential greater than − 0.414 V, up to cadmium in the electrochemical series, can displace hydrogen from water. This displacement occurs when the metal forms soluble hydroxides or oxides as reaction products. The reactivity of metals in displacing hydrogen is highest for those at the top of the electrochemical series. However, the solubility of the formed products plays a crucial role. If the reaction leads to the formation of poorly soluble compounds, particularly common with metal hydroxides, the metal surface becomes passivated. This passivation occurs because the poorly soluble compounds act as a protective layer, preventing further reaction between the metal and water. Metals located in the electrochemical series between magnesium and cadmium typically do not react with water at normal temperatures due to the formation of insoluble hydroxides^61^.

The electrode potential plays a crucial role in determining the interaction of metals with water by governing the redox reactions at the metal-water interface. Metals with lower electrode potential than water can reduce water molecules, producing hydrogen gas and hydroxide ions. This reaction is particularly relevant in neutral or slightly basic environments where the concentration of hydroxonium ions (H_3_O^+^) is low.

The electrode potential also influences the formation of protective oxide or hydroxide layers on the metal surface. Metals with high electrode potential are more likely to form stable oxide films, inhibiting further water reactions and reducing wear. Conversely, metals with lower potential are prone to ongoing reactions, increasing hydrogen generation and potential hydrogen embrittlement. Thus, controlling electrode potential through material selection and design is critical to minimizing wear and degradation in water-exposed systems.

Understanding the interaction between water and metal surfaces in road construction machinery is essential for mitigating wear and extending the lifespan of critical components. Water acts as both a corrosive agent and a source of hydrogen atoms through electrochemical reactions. These hydrogen atoms can diffuse into metal surfaces, leading to hydrogen embrittlement and accelerated material degradation. Additionally, the presence of water exacerbates the formation of corrosion products and wear debris, further damaging metal surfaces and adjacent components. This interaction is particularly significant in wet or damp operating conditions, where the combined effects of corrosion and hydrogen diffusion can severely compromise the integrity and performance of machinery. By analysing these interactions, researchers can develop advanced materials, protective coatings, and maintenance strategies to improve the durability and efficiency of road construction equipment.

Passivation is a phenomenon where the surface of a metal becomes less reactive due to the formation of a protective oxide or hydroxide layer. This thin, stable film acts as a barrier, preventing further interaction between the metal and its environment, including water. For example, metals like chromium and aluminum form adherent oxide layers that inhibit water-induced corrosion and hydrogen generation. The effectiveness of passivation depends on factors such as the metal’s chemical composition, environmental conditions, and the stability of the oxide layer. Passivated metals exhibit significantly reduced reactivity with water, as the protective layer limits direct contact with the metal surface. However, when the passivation layer is disrupted or destroyed, the metal becomes highly reactive, allowing processes such as hydrogen evolution and corrosion to occur rapidly. Understanding passivation is crucial for designing materials that can withstand harsh environments and maintain their integrity over time.

The interaction of steel 35KhNL with hydrogen is significantly influenced by its chemical composition, with each element playing a distinct role. Iron (Fe), the primary component, readily absorbs hydrogen, which diffuses through its crystal lattice as protons, contributing to hydrogen embrittlement. Chromium (Cr) and nickel (Ni) enhance resistance by forming stable hydrides and altering hydrogen solubility, though their effectiveness varies with temperature. Manganese (Mn) exhibits temperature-dependent behaviour, with hydrogen solubility decreasing at lower temperatures and increasing above 500 °C. Silicon (Si) provides limited interaction with hydrogen, whereas sulfur (S) facilitates the formation of hydrogen sulfide, further promoting material degradation at elevated temperatures. Copper (Cu), though present in small amounts, affects thermal conductivity and hydrogen occlusion under high-temperature conditions. The synergistic effects of these elements define the steel’s susceptibility to hydrogen embrittlement and its overall durability, underscoring the importance of compositional control in mitigating hydrogen-induced wear.

Interestingly, many metals possess a strong oxide film on their surface, which serves a protective function. This oxide film is a significant factor in limiting the reactivity of certain metals with water. For instance, aluminium theoretically has the potential to react vigorously with water, but this does not occur due to the presence of a robust, water-insoluble oxide film. This oxide film on aluminium’s surface renders it resistant to water and steam. However, when this protective film is disrupted or destroyed, aluminium becomes highly reactive with water, liberating hydrogen gas.

**Table:** 

2	Al + 3H_2_O – 3e = Al(OH)_3_ + 3 H^+^
3	2H_2_O + 2e = H_2_ + 2OH^-^
2Al + 6H_2_O + 6H_2_O = 2Al(OH)_3_ + 3H_2_ + 6 H^+^ + 6OH^-^ 2Al + 6H_2_O = 2Al(OH)_3_ + 3H_2_	

Passivation refers to the phenomenon where a metal’s oxidation is significantly inhibited due to the formation of a protective oxide or salt film on its surface. This protective layer acts as a barrier, preventing further reaction of the metal with its environment and providing resistance against corrosion. Passivation is crucial in enhancing certain metals’ corrosion resistance under specific conditions. Under atmospheric conditions, several metals exhibit passivation by forming stable and protective films. Some notable metals that commonly undergo passivation include chromium, nickel, aluminium, cadmium, and zinc. Let us delve into the passivation characteristics of these metals:

- Chromium: Chromium forms a thin and stable oxide layer (chromium oxide, Cr_2_O_3_) on its surface, which protects against further oxidation and corrosion. This passivation is essential for the corrosion resistance of stainless steel, which contains a significant amount of chromium.
- Nickel: Nickel, when exposed to air, develops a passive oxide layer (nickel oxide, NiO) on its surface. This oxide film enhances nickel’s resistance to corrosion and oxidation.
- Aluminium: Aluminium is known for its rapid formation of a protective oxide layer (aluminium oxide, Al_2_O_3_) upon exposure to air. This oxide film is compact, adherent, and provides effective protection against corrosion, contributing to the overall durability of aluminium^62,63^.
- Cadmium: Cadmium undergoes passivation by forming a thin layer of cadmium oxide (CdO) on its surface. This oxide film acts as a protective barrier, preventing further cadmium corrosion.
- Zinc: Zinc readily forms a protective layer of zinc oxide (ZnO) or zinc carbonate (ZnCO_3_) when exposed to air. This passivation is vital for galvanised steel’s corrosion resistance, where a zinc layer is applied to protect the underlying iron or steel from corrosion.

The paper contributes significantly to understanding the adsorption and diffusion of hydrogen in nickel and other metals by detailing the mechanisms of hydrogen interaction at both the surface and subsurface levels. In nickel, the study reveals that hydrogen adsorption leads to the formation of a hydrogen-nickel system characterized by a metallic bond type composition with a high hydrogen-to-nickel ratio. This interaction enhances hydrogen solubility and diffusion within the crystal lattice. Furthermore, the research identifies critical factors such as temperature, concentration, and lattice structure that influence hydrogen mobility and embrittlement across different metals. By examining these phenomena, the study provides a comprehensive framework for analyzing hydrogen behavior in metallic systems, offering insights that are essential for developing strategies to mitigate hydrogen-induced wear and optimize material performance in demanding environments.

The passivation process plays a critical role in preserving the integrity and longevity of metals in various applications, especially in contexts where exposure to corrosive environments is a concern.

**Table 2:** Chemical composition of friction lining materials.

	Friction lining code								
	Element content, %								
	N_free_	S	Al	Cu	Fe	Si	Zn	Pb	Ni
G	16.40	3.70	3.33	7.66	3.69	0.64	2.78	3.10	0.260
B	24.20	2.95	3.95	3.34	14.90	1.08	2.23	0.08	0.260
C	18.10	-	0.84	11.80	27.40	-	3.28	0.13	-
A	22.70	-	1.97	5.84	14.30	1.12	3.67	0.16	0.023
H	19.60	-	0.24	5.13	19.50	0.34	1.10	0.01	0.004
D	19.10	-	0.13	11.77	35.20	0.29	3.52	0.05	0.008
J	22.30	-	0.31	0.23	30.90	0.21	0.07	2.08	0.001
K	**-**	**-**	0.50	9.50	32.70	0.28	2.68	2.40	-
L	**-**	**-**	0.36	10.50	34.00	0.29	2.97	2.66	**-**
E	19.4	19.4	0.67	8.23	19.4	0.11	2.41	0.09	0.01

	Friction lining code								
	Element content, %								
	Ti	Sb	Ba	Ca	K	Mn	Mg	Na	Sr
G	**-**	**-**	**-**	**-**	**-**	**-**	**-**	**-**	**-**
B	**-**	**-**	**-**	**-**	**-**	**-**	**-**	**-**	**-**
C	0.140	**-**	**-**	**-**	**-**	**-**	**-**	**-**	**-**
A	0.140	**-**	**-**	**-**	**-**	**-**	**-**	**-**	**-**
H	0.060	**-**	**-**	**-**	**-**	**-**	**-**	**-**	**-**
D	0.020	**-**	**-**	**-**	**-**	**-**	**-**	**-**	**-**
J	0.060	**-**	**-**	**-**	**-**	**-**	**-**	**-**	**-**
K	0.040	4.50	0.84	**-**	**-**	**-**	**-**	**-**	**-**
L	0.040	4.90	0.58	**-**	**-**	**-**	**-**	**-**	**-**
E	0.002	**-**	2.88	2.88	0.04	0.13	0.10	0.05	0.02
F	0.080	**-**	2.87	0.36	0.21	0.17	0.30	0.05	0.03

Moving to Table 2 the chemical composition of friction lining materials is detailed. The choice of materials for friction linings is a critical factor. particularly in applications like brakes^64,65^. This table provides information on how these materials interact with water and hydrogen—essential considerations for ensuring optimal performance and longevity of the friction pair. Table 3 guides the selection of components for the metal-polymer friction pair. The emphasis here is on choosing materials whose electrode process potential in a neutral medium is lower than water’s. Striking this balance is vital to prevent undesirable reactions that could compromise the effectiveness of the friction pair. However, an issue is identified in the overestimated nickel (Ni) ratio of 7000.0. it is indicating a potential error in the selection of the percentage composition. Addressing this discrepancy is crucial to aligning with specified intervals and maintaining the desired properties of the friction lining material.

**Table 3:** The ratio of the chemical composition of the materials of the friction pair *metal-polymer*.

Ratio						
Materials						
Fe		Si	Cu	Mn	Ni	S
min	26.1	1.8	1.3	5.3	7000.0	0.018
max	2.8	0.39	0.25	3.0	3.5	0.02

## Interaction of atoms in the hydrogen-metal system

According to the basic principles of nonequilibrium thermodynamics, the flux density j of diffusing atoms under isothermal conditions is proportional to the gradient of their chemical potential $\nabla \upmu :$

8 $$\:j=-L\nabla\:\mu\:=-L\frac{\partial\:\mu\:}{\partial\:n}\nabla\:n$$

Where:

*L* – isothermal kinetic coefficient;

$$\nabla$$ - Hamilton operator.

*n* – volume concentration of diffusing atoms.

The second equality assumes that the chemical potential depends only on the concentration but not its derivatives. Which is valid for “smooth” concentration inhomogeneities. Formula (8) is Fick’s first law j = -*D(c)*$\nabla$*n*. where *с* = *nΩ*. *c*$\le 1$ - hydrogen concentration determined by the number of interstices. *Ω* – volume per one internode; diffusion coefficient *D(c) = LΩ∂µ/∂c*includes (through the chemical potential) the entropy contribution and the contribution due to the interaction of diffusing atoms (this also consists of the electronic contribution). In the simplest case. The maximum concentration approximation can be introduced^66^. This approximation assumes that the hydrogen concentration cannot exceed a certain “maximum” value *с*_*m*_ (for Н and Fe. for example, under normal conditions с_m_≈ 0.6). Then, the electron contribution can be neglected throughout this “allowed” concentration region since it is small^67–69^. In addition, for the H – H interaction. we accept the equivalent field approximation. Then, from (1) follows the concentration dependence of the KD

9 $$D\left( c \right) = D\left[ {1 + \frac{U}{{KT}}c\left( {1 - \frac{c}{{c_{m} }}} \right)} \right],$$

Where:

*D* – diffusion coefficient in a dilute solution (at *с* → 0).

*U* - an energy parameter that includes the energy of hydrogen atoms’ electrochemical and elastic interaction.

From (2), it follows that at *U* < 0 (interaction has the character of attraction), for each value of c. there is a steady temperature *Т*_ζ_. with which *D*(*c*) = 0:

10 $$T_{\varsigma } = - \frac{U}{k}c\left( {1 - \frac{c}{{c_{m} }}} \right).$$

At Т < *Т*_ζ_. the effective KH *D*(c) becomes negative. and the motion of atoms. strictly speaking. can no longer be called diffusion since it increases inhomogeneities. Nevertheless, since Fick’s law is formally fulfilled. The “diffusion” terminology has been preserved even when Т < *Т*_ζ_ and the corresponding transfer of atoms at *D*(c) < 0 is known as “upward diffusion”. According to what has been said, we will deliberate Fick’s law as an effective equivalent diffusion coefficient. Equation (3) coincides with the equation known in the theory of spinodal decomposition, which determines the boundary of the instability of a solid solution for minor fluctuations. The spinodal at Т < *Т*_ζ_. the solid solution becomes unstable and decomposes into two phases^70,71^, differing in the content of interstitial atoms. The solution of the diffusion equation with effective KD H (10) showed that as the temperature decreases, hydrogen more and more slowly penetrates from the gas phase into the volume of the hydride-forming metal, localizing mainly in a thin surface layer. This conclusion is in qualitative agreement with the experimental results for steel.

At higher concentrations, new effects are possible. Thus, it was experimentally shown in [11.12] that at *c* > 0.6, the concentration dependence of the effective hydrogen condensate factor in steel has a maximum, the relative value of which increases with decreasing temperature.

**Fig. 4:**
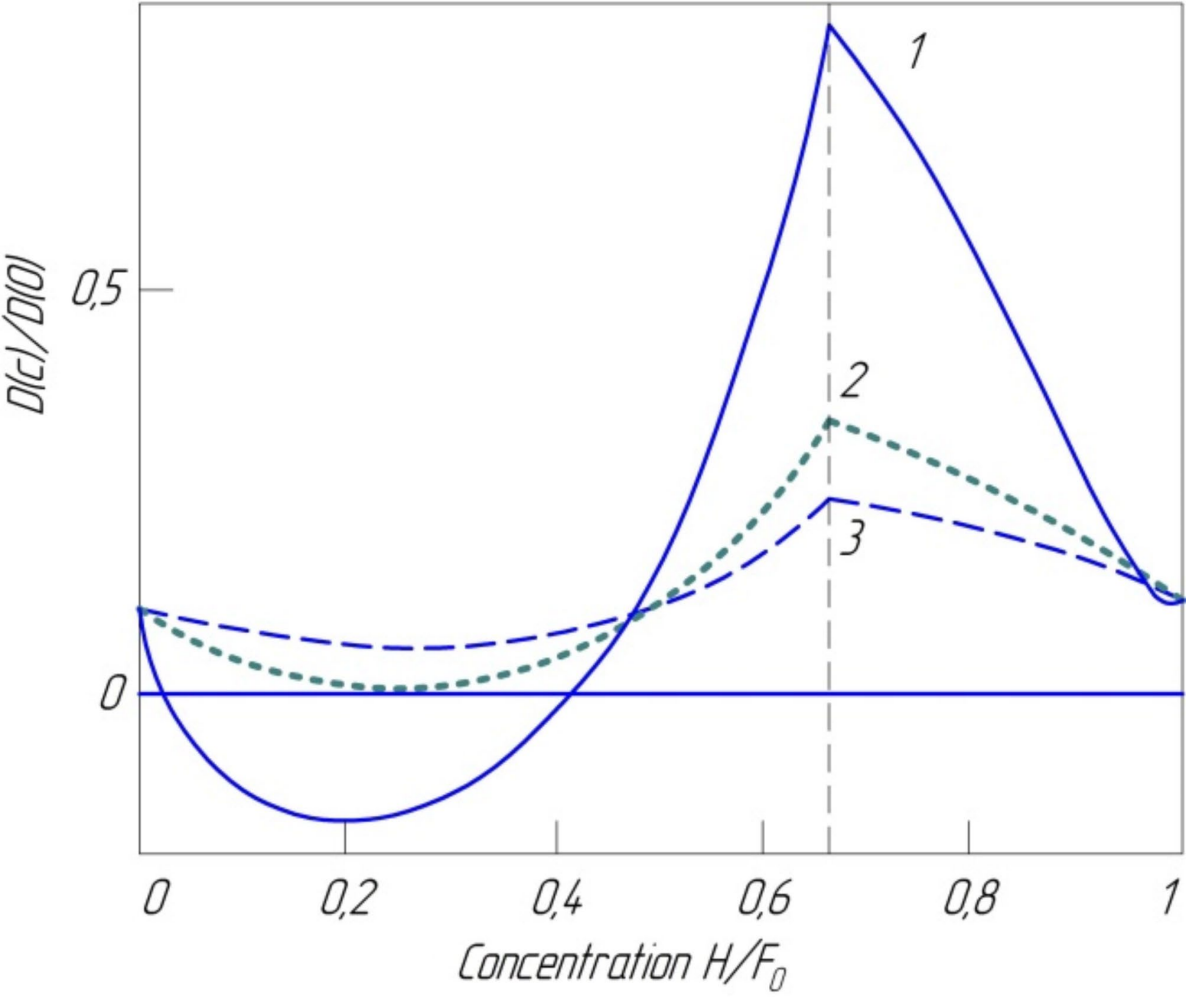
Concentration dependence of the diffusion coefficient of hydrogen in iron: 1 – T = 500 K; 2–880 K; 3–1000 K.

In the article, the concentration dependence of KD H in Fe was obtained in a wide range of concentrations within the quasi-chemical approximation, considering the electronic and vibrational contributions to the chemical potential (Fig. 4; the break in the curves at the maximum point *c* = 0.656 is due to the form of the empirical dependence of the electronic chemical potential given in eleven]). The curves in Fig. 4 qualitatively correctly describe the observed features of the concentration dependence of the effective KD in Fe: the KD decreases to negative values ​​with increasing concentration on the one hand, and the presence of a maximum at *c* > 0.6. on the other. The value *D*(c), corresponding to the critical temperature *T*_c_ = 750 K, vanishes at *c* = 0.25. This value coincides with the experimentally established value of the required concentration *c*_c_= 0.250 ± 0.005^67^. The presence of a maximum on the *D*(c) curve at *c* > 0.6 is naturally explained by a sharp increase in the electronic chemical potential in this concentration range and by the fact that at *c* → 1 *D*(c) → *D*. Indeed, with almost complete filling of the interstices, as well as at low concentrations, the interaction of diffusing atoms does not play a significant role^72^.

The key findings of the research indicate that hydrogen behaviour in metal surfaces under high temperatures and pressure is characterised by complex interactions with the material’s crystal lattice. Hydrogen atoms diffuse into the metal as protons, increasing the diffusion rate as temperature and pressure rise. However, at elevated concentrations, hydrogen interactions with the lattice and other hydrogen atoms lead to structural instability, including embrittlement and localised material degradation. The study also highlights a critical concentration beyond which diffusion dynamics shift, contributing to reduced material integrity. Additionally, temperature gradients influence hydrogen localisation, often confining hydrogen to surface layers and exacerbating wear in high-stress applications. These findings underscore the importance of controlling environmental conditions and material properties to mitigate hydrogen-induced damage, particularly in braking systems and heavy machinery applications.

Polymers play a vital role in wear resistance within metal-polymer friction pairs, particularly in brake systems, by acting as a cushioning layer that reduces direct metal-to-metal contact and dissipates mechanical stress. Commonly used polymers, such as epoxy resins, polyurethane, and phenolic resins, are engineered for their high thermal stability, mechanical strength, and low wear rates under dynamic operating conditions. However, their interactions with hydrogen can significantly influence their performance. Hydrogen diffusion into the polymer matrix can disrupt intermolecular bonds, leading to embrittlement, reduced elasticity, and diminished load-bearing capacity. This degradation is exacerbated in high-temperature environments, where polymer structures become more susceptible to chemical and mechanical alterations. Phenolic resins, widely used in brake systems, demonstrate superior hydrogen resistance due to their thermoset structure, which offers enhanced stability. A deeper understanding of these interactions is critical to optimizing the composition of friction pairs, balancing the wear resistance of polymers with the durability of the metal components to achieve long-term performance in hydrogen-exposed environments.

Let us consider the kinetic effects of the interaction of interstitial subsystems in a metal matrix. First, let us briefly discuss the results associated only with the possible occupation of interstices. It is considered an ideal multicomponent lattice gas of noninteracting interstitial atoms. In an ideal lattice gas, a kind of “Pauli principle” prohibits two interstitial atoms from simultaneously being in the same interstices, causing the appearance of corresponding correlations in the filling of interstices. In particular, the probability of diffusion jumps of atoms of a given component depends on the degree of filling of interstices with atoms of all components of the lattice gas. The diffusion flux of atoms of a certain type, even in the absence of interaction, depends not only on its concentration gradient but also on the concentration gradients of atoms of other types^73^:

11 $$j_{i} = - \sum\limits_{{\lambda = 1}}^{m} {D_{{i\lambda }} \nabla n_{\lambda } ,\quad i = 1,2, \ldots ,m,}$$

Here, the components $D_{{i\lambda }}$ matrices of diffusion coefficients turn out to depend on the concentrations of all *m* components:

12 $$D_{{i\lambda }} = D_{i} \left[ {c_{i} + \delta _{{i\lambda }} \left( {1 - c} \right)} \right].$$

The formula (12) *D*_*i*_ – diffusion coefficient *i*- th component in a dilute interstitial alloy.

$$c = \sum\limits_{{i = 1}}^{m} {c_{i} } .\;\delta _{{ik}} {\text{ - Kronker}}'{\text{s}}\;{\text{symbol}}.$$

*δ*_*ik*_ – Kronker’s symbol.

It is vital that, as the concentrations increase. The off-diagonal components of the CD matrix increase while the diagonal ones decrease. Therefore, at high degrees of interstitial filling, the off-diagonal components of the diffusion tensor can become equal to the diagonal ones. We emphasize that a nontrivial result manifests itself here. In contrast to^74^ when one simple physical reason is considered – the effect of interstitial occupancy. Under typical experimental conditions, constant partial pressures of the components in the gas phase are usually maintained on both sides of the sample (membrane). The analysis shows that changing the concentrations of other components on the inlet and outlet sides of the membrane makes it possible to change the flux of atoms of a given type over a wide range. It turns out that the flux of atoms of a given component is not equal to zero even when its concentration gradient is absent.

The following effect is considered an illustration of the technological importance of the obtained relations. The tritium atoms are randomly distributed in the membrane, the desorption of which is difficult at low tritium concentrations. If deuterium is supplied to the inlet side of the membrane, then, according to (4), a diffusion flux of tritium arises caused by the deuterium concentration gradient and is directed toward the outlet side of the membrane. This leads to a decrease in the tritium concentration in the sample, and over time, almost complete degassing of the sample from tritium occurs. It is easy to see that this effect can be helpful for thermonuclear power engineering, where the technological problem of collecting and utilizing radioactive tritium is critical from a technical and environmental point of view. Taking into account the interaction of interstitial atoms in a multicomponent concentrated lattice gas in the framework of the statistical approach for the case of diffusion along O-interstices of the BCC metal lattice in the usual approximations of the diffusion theory led to expression (4) with the KD matrix

13 $$D_{{i\lambda }} = D_{i} \left[ {c_{i} + \left( {1 - c} \right)\left( {\delta _{{i\lambda }} + \frac{{5u_{{i\lambda }} }}{{kT}}c_{i} } \right)} \right]\exp \left( { - \sum {\nu _{{i\lambda }} c_{\lambda } /kT} } \right).$$

Here *w*_iλ_ – interaction energy of two neighboring interstitial atoms of the components *i* and *k*. *ν*_*iλ*_ - energy parameters.

### Diffusion dynamics

The concentration-dependent diffusion coefficient of hydrogen in iron provides valuable insights into hydrogen transport mechanisms and their effects on material behaviour. At low hydrogen concentrations, diffusion is primarily driven by free interstitial movement, resulting in an increasing diffusion coefficient. However, as the concentration surpasses a threshold, hydrogen-hydrogen interactions and changes in the electronic chemical potential impede mobility, leading to a decline in the diffusion coefficient. This non-linear relationship highlights the complex interplay between atomic interactions and concentration effects, critical to understanding hydrogen-induced phenomena such as embrittlement and wear. These findings are essential for developing strategies to optimise material performance and durability in hydrogen-exposed environments.

The “upward diffusion” phenomenon occurs when the effective diffusion coefficient becomes negative, causing atoms to migrate toward regions of higher concentration rather than dispersing to lower concentration areas. This counterintuitive behavior is observed at temperatures below a critical threshold, known as TζT_\zetaTζ​, and is reminiscent of spinodal decomposition in solid solutions. In this state, the material becomes thermodynamically unstable, amplifying concentration inhomogeneities and potential phase separation.

In material science, upward diffusion has significant implications, particularly in understanding and controlling microstructural evolution. It provides insights into the conditions that lead to phase instability and localised concentration changes, affecting mechanical properties, durability, and material performance. By leveraging this phenomenon, researchers can design materials with tailored microstructures for specific applications, optimise processing conditions, and mitigate undesirable effects such as embrittlement or premature failure. This understanding is especially crucial in fields like metallurgy, semiconductors, and energy storage, where precise control over diffusion processes is critical.

## Results and discussion

Understanding the intricate linkage between atoms in the hydrogen-metal system is crucial for various technological applications, from materials science to energy production. This section discusses the results obtained from our investigation and their implications in greater detail.

###  Diffusion dynamics and Fick’s law

The foundational principle of nonequilibrium thermodynamics. as expressed by the equation =−j = − L∇µ, is a cornerstone in understanding diffusion phenomena within metal lattices. This equation. Analogous to Fick’s first law, it offers a robust theoretical framework for elucidating the intricate dynamics of atom movement in response to concentration gradients. By quantifying the flux density of diffusing atoms relative to the gradient of their chemical potential, we gain profound insights into the fundamental mechanisms that govern diffusion processes in diverse material systems^75–77^. At its core, the equation j = − L∇µ underscores the essential role of concentration gradients in driving the movement of atoms within a metal lattice. The flux density (j) represents the rate at which atoms migrate through the lattice per unit area. At the same time, ∇µ signifies the gradient of the chemical potential, which encapsulates the driving force for diffusion. This gradient arises from variations in the concentration of diffusing species across space, setting the stage for atom migration from regions of higher chemical potential to those of lower potential. In essence. The equation j = − L∇µ provides a quantitative link between the macroscopic phenomenon of diffusion and the underlying microscopic processes governing atomic motion. The kinetic coefficient (L) encapsulates the material-specific properties influencing the diffusion rate, such as atomic mobility and interaction energies. Meanwhile, the gradient of the chemical potential reflects the thermodynamic driving force for diffusion arising from differences in atomic concentration^78–80^.

We can discern the underlying mechanisms that drive diffusion processes in metal lattices by examining the relationship between flux density and the chemical potential gradient. For instance, temperature, pressure, or composition variations can alter the chemical potential gradient, influencing the rate and direction of atomic migration. Moreover, the kinetic coefficient (L) provides insights into diffusion efficiency within a given material. Shedding light on defect density, crystal structure, and interatomic interactions. Furthermore. The analogy to Fick’s first law underscores the universality of diffusion phenomena across different physical systems. They highlight the fundamental nature of atomic motion in response to concentration gradients. Whether in metallic alloys, semiconductors or biological tissues, the principles encapsulated by j = − L∇µ offer a unifying framework for understanding diffusion processes and their implications for material properties and behaviour^81,82^.

The research findings demonstrate that temperature profoundly impacts the effective diffusion coefficient of hydrogen in iron. At higher temperatures, the diffusion coefficient generally increases due to enhanced atomic mobility and reduced resistance within the metal lattice. However, a critical phenomenon arises at temperatures below the threshold TζT_\zetaTζ​, where the effective diffusion coefficient becomes negative, leading to “upward diffusion” — the migration of hydrogen atoms toward regions of higher concentration. This behavior signals a breakdown in the thermodynamic stability of the material and is analogous to spinodal decomposition in solid solutions. Understanding this temperature-dependent behavior is essential for predicting and mitigating hydrogen-induced material degradation, as it highlights the conditions under which hydrogen mobility may accelerate embrittlement or other structural instabilities.

### Temperature effects and phase transitions

One of the significant findings of our analysis is the identification of a critical temperature, denoted as T_ζ_, below which the effective diffusion coefficient exhibits a peculiar behaviour, becoming negative and giving rise to what is termed “upward diffusion.” This phenomenon is reminiscent of spinodal decomposition observed in solid solutions and has profound implications for understanding diffusion dynamics in materials. At temperatures T < T_ζ_, the effective diffusion coefficient takes on negative values, indicating a reversal of atomic migration direction. This unexpected behaviour challenges conventional notions of diffusion, where atoms typically move from regions of higher concentration to lower concentration. Instead, under the influence of temperature below T_ζ_, atoms migrate towards regions of higher concentration, exacerbating concentration inhomogeneities within the material^83^.

The analogy to spinodal decomposition, a phenomenon observed in solid solutions undergoing phase separation, provides valuable insights into the underlying mechanisms driving upward diffusion. In spinodal decomposition, a solid solution becomes thermodynamically unstable below a critical temperature, leading to the spontaneous formation of distinct phases with differing compositions. Similarly, the transition to negative effective diffusion coefficients below T_ζ_ signals a breakdown in the equilibrium state of the material, resulting in the amplification of concentration gradients and the onset of phase separation-like behaviour.

Understanding the temperature dependence of diffusion dynamics, particularly in the context of phase transitions, is essential for predicting and controlling the evolution of material microstructures. By elucidating the conditions under which upward diffusion occurs, researchers can gain valuable insights into the mechanisms governing phase separation and concentration patterns in materials. This knowledge is particularly relevant for designing materials with tailored properties, where precise control over microstructural evolution is crucial for achieving desired performance characteristics^84,85^. Furthermore, the observation of negative effective diffusion coefficients below T_ζ_ highlights the intricate interplay between temperature and concentration gradients and atomic mobility in materials. By probing the temperature dependence of diffusion behaviour, researchers can uncover new avenues for manipulating material properties and engineering novel functionalities. For instance, by precisely controlling the temperature environment during material processing, it may be possible to induce desired phase transitions and microstructural transformations, leading to the development of advanced materials with enhanced performance and functionality.

### Concentration dependence and maximum effect

The concentration dependence of the effective diffusion coefficient unveils a fascinating interplay of factors, particularly evident in iron (Fe), where an intriguing maximum is observed at higher concentrations (c > 0.6). This phenomenon, known as the maximum effect, represents a crucial aspect of diffusion behaviour that arises from the complex interactions between concentration gradients and electronic properties within the material^86,87^. Understanding the underlying mechanisms behind this maximum effect is essential for elucidating the nonlinear nature of diffusion processes and exploring potential strategies for optimizing material properties through concentration modulation. At lower concentrations, the diffusion coefficient typically follows a predictable trend, where an increase in concentration leads to a corresponding increase in the diffusion rate. However, a deviation from this trend occurs as the concentration approaches higher values, resulting in the emergence of a maximum in the concentration dependence of the diffusion coefficient. This deviation is attributed to the intricate interplay between diffusion-related processes and electronic properties inherent to the material.

In the case of iron, this maximum effect can be attributed to several underlying factors. Firstly, at higher concentrations, the interactions between diffusing atoms become more pronounced, leading to increased electron density and alterations in the material’s electronic structure. These changes in electronic properties can significantly influence diffusion behaviour, causing deviations from the expected linear relationship between concentration and diffusion coefficient. Moreover, the maximum effect in iron highlights the importance of considering electronic contributions in diffusion processes^88^. Unlike simple diffusion models that solely focus on concentration gradients, incorporating electronic effects provides a more comprehensive understanding of diffusion behaviour, especially in materials with significant electronic interactions. By accounting for electronic contributions, researchers can better predict and explain observed deviations from ideal diffusion behaviour, such as the emergence of maximum effects at higher concentrations.

Furthermore, the presence of a maximum in the concentration dependence of the diffusion coefficient suggests intriguing possibilities for optimizing material properties through concentration modulation. By strategically manipulating the concentration of diffusing atoms, either through alloying or doping techniques, it may be possible to tailor the diffusion behaviour of the material to achieve the desired characteristics. For example, controlling the concentration profile near the surface of a material could enhance its performance in specific applications where diffusion processes play a critical role, such as catalysis or semiconductor device fabrication^89,90^.

### Multicomponent Lattice gas and correlation effects

Extending our analysis to encompass a multicomponent lattice gas of noninteracting interstitial atoms unveils a rich tapestry of correlation effects that intricately shape diffusion phenomena within the lattice structure. Unlike the simpler scenarios involving single-component systems, the presence of multiple atomic species introduces a myriad of interdependencies and correlations among different types of atoms occupying the interstitial sites^91,92^. These correlations manifest as subtle yet profound influences on diffusion dynamics, necessitating a deeper understanding of accurate modelling of diffusion processes in complex material systems. One of the key insights derived from our extended analysis is the emergence of correlation effects in filling interstices within the lattice. Unlike in single-component systems where atoms occupy interstitial sites independently, in a multicomponent lattice gas, the occupancy of interstices becomes intricately correlated among different atomic species. This phenomenon is akin to a “Pauli principle” in quantum mechanics, albeit in a classical context, where the presence of one type of atom affects the probability of finding another type of atom in nearby interstices.

Consequently, the diffusion flux of atoms is not solely determined by the concentration gradient of their type but also by those of other types, giving rise to complex interdependencies that significantly influence diffusion behaviour^93^. Moreover, these correlation effects are not confined to interactions between atoms of the same type but extend to interactions between different atomic species within the lattice. The diffusion flux of a particular type of atom becomes intricately linked to the concentration gradients of all other atoms present in the lattice, reflecting the interconnected nature of diffusion processes in multicomponent systems. This interplay between different atomic species adds another layer of complexity to diffusion dynamics and underscores the importance of accounting for cross-species interactions in diffusion modelling.

Understanding these correlation effects is paramount for accurately modelling diffusion processes in complex material systems. Traditional diffusion models that neglect these interdependencies may fail to capture the nuanced behaviour exhibited by multicomponent systems, leading to inaccuracies in predicting diffusion rates and pathways. By incorporating knowledge of correlation effects into diffusion models, researchers can enhance the fidelity of their simulations and gain deeper insights into the underlying mechanisms governing diffusion phenomena in diverse materials. Furthermore, elucidating correlation effects opens up new avenues for tailored design and engineering of materials with desired diffusion properties^94,95^. By manipulating the composition and arrangement of atomic species within a lattice, it may be possible to modulate diffusion behaviour in a controlled manner, enabling the development of materials with enhanced transport properties or tailored diffusion pathways. This represents a promising direction for future research in materials science and engineering, where the ability to control diffusion processes precisely holds immense potential for advancing technological frontiers.

### Technological implications and applications

The insights derived from our theoretical analysis carry profound implications for various technological applications, with particularly notable ramifications in thermonuclear power engineering. By harnessing concentration gradients of other atoms, such as deuterium, we unlock the potential to manipulate the diffusion flux of specific species, notably tritium, thus enabling their effective removal from metal samples. This capability presents a transformative solution to addressing critical technological challenges^96,97^, foremost among them being the collection and utilization of radioactive tritium in a safe and efficient manner. In thermonuclear power engineering, the ability to control the diffusion flux of tritium holds immense significance. Tritium, a radioactive isotope of hydrogen, is a key component in fusion reactions and is used in various nuclear applications. However, managing tritium poses significant challenges due to its radioactive nature and potential hazards to human health and the environment. One of the primary concerns is the accumulation of tritium in metal structures, which can occur during operation or as a byproduct of nuclear processes.

Our theoretical framework offers a novel approach to address this challenge by exploiting the principles of diffusion and concentration gradients. By introducing deuterium, which shares similar diffusion properties with hydrogen isotopes, into the system, we can effectively manipulate the diffusion flux of tritium. This is achieved by establishing concentration gradients of deuterium, which in turn induce a directional flow of tritium away from the metal samples. This innovative strategy has several key advantages. Firstly, it provides a mechanism for actively removing tritium from metal structures, thereby reducing the risk of contamination and mitigating potential safety concerns associated with tritium exposure. Secondly, the proposed method offers a cost-effective and scalable solution by leveraging existing infrastructure and processes for handling deuterium, such as gas handling systems and purification techniques.

Additionally, the ability to control the diffusion flux of tritium enables precise management of tritium levels within metal samples, ensuring compliance with regulatory standards and safety protocols. Moreover, the implications extend beyond nuclear applications, encompassing a wide range of industrial processes where removing specific species from metal samples is desirable. For example, in the semiconductor industry, the precise control of impurity concentrations in metal substrates is critical for ensuring the quality and performance of electronic devices. Adapting our approach makes it possible to tailor diffusion processes to meet the unique requirements of various manufacturing processes, thereby enhancing product quality and efficiency.

### Interaction energies and modulation of diffusion dynamics

A deeper investigation into the interaction energies between neighbouring interstitial atoms of different components within the lattice unveils a nuanced understanding of their influence on diffusion dynamics. These interaction energies, coupled with associated parameters, intricately dictate the propensity of atoms to diffuse within the lattice and consequently mould the overarching diffusion behaviour exhibited by the material system. By meticulously accounting for these interaction energies, our theoretical framework unravels the complex interplay between atoms and offers profound insights into the fundamental mechanisms governing diffusion processes^98–101^. Moreover, it furnishes a robust foundation for anticipating and manipulating diffusion behaviour across a spectrum of material systems, catalysing technological advancements. The interaction energies between neighbouring interstitial atoms serve as pivotal determinants in shaping the diffusion kinetics within a lattice structure. These energies govern the strength of the bonds formed between atoms, consequently influencing the likelihood of atomic migration within the lattice.

Moreover, they mediate the extent of atomic interactions, such as repulsion or attraction, further modulating diffusion dynamics. By scrutinizing these interaction energies, our theoretical framework discerns subtle nuances in the diffusion process, elucidating how variations in energy landscapes give rise to distinct diffusion behaviours observed in different material systems.

Furthermore, the parameters linked to these interaction energies play a crucial role in fine-tuning diffusion dynamics. These parameters encapsulate factors such as atomic size, electronic configuration, and lattice symmetry, all of which influence diffusion behaviour. Through a comprehensive analysis of these parameters, our theoretical framework unveils the underlying mechanisms dictating diffusion processes, thereby offering predictive capabilities for discerning how material properties manifest in diffusion phenomena. By integrating these insights into our theoretical framework, we gain a holistic understanding of diffusion behaviour that transcends conventional models. Rather than treating diffusion as a simplistic process governed solely by concentration gradients, our approach acknowledges the intricate interplay of atomic interactions and energy landscapes. This nuanced perspective enhances our comprehension of diffusion phenomena and equips us with powerful tools for engineering materials with tailored diffusion properties^102–104^.

Moreover, the predictive capabilities afforded by our theoretical framework hold immense value for diverse technological applications. We can optimize material design and processing strategies to achieve desired performance metrics by accurately predicting diffusion behaviour in complex material systems. Whether in developing advanced alloys, designing high-performance electronic devices, or optimising chemical processes, our framework serves as a guiding beacon for harnessing diffusion phenomena to drive technological innovation forward.

## Industries benefiting from the research

The study highlights several strategies for mitigating wear-induced challenges in road construction equipment. Regular maintenance and timely replacement of worn components are critical to reducing the accumulation of wear-induced damage. Advanced material selection, such as corrosion-resistant alloys and coatings, helps combat the effects of hydrogen embrittlement and corrosion in wet environments. Implementing thermal stabilisation techniques ensures uniform temperature distribution, minimising localised stresses that exacerbate wear. Additionally, improving the design of critical components, such as brake systems, to enhance durability and resistance to mechanical stress is emphasised. These strategies collectively aim to extend the operational lifespan of construction machinery while maintaining efficiency and safety under demanding conditions.

- Automotive Manufacturers: The research findings benefit leading automotive companies like Toyota, Ford, Volkswagen, and General Motors significantly. By enhancing the performance and safety of brake friction pairs, these manufacturers can improve their vehicles’ overall quality and reliability.
- Aerospace Companies: Major players in the aerospace industry, including Boeing, Airbus, Lockheed Martin, and SpaceX, can utilize insights from the research to develop more durable materials for critical aircraft components. This could lead to advancements in aerospace technology, improving safety and efficiency in air travel.
- Manufacturing Equipment Suppliers: Companies such as Siemens, Bosch, and Caterpillar, which provide manufacturing equipment across various industries, can apply the research findings to enhance the durability and efficiency of their products. This could result in more reliable and cost-effective manufacturing processes for various applications.
- Renewable Energy Companies: Companies involved in hydrogen fuel cell technology, such as Ballard Power Systems, Plug Power, and Bloom Energy, can benefit from advancements in hydrogen wear reduction. By improving the durability of fuel cell materials, these companies can enhance the reliability and performance of hydrogen-based energy systems, contributing to the growth of renewable energy solutions.
- Materials Science and Engineering Firms: Organizations specializing in research and development, including Corning Incorporated, DuPont, and BASF, can leverage the research findings to improve material performance across various applications. This could lead to the development of more advanced and sustainable materials for the electronics and construction industries.

The findings of this research have significant potential benefits for automotive manufacturers and other industries, particularly in enhancing the durability and efficiency of components exposed to hydrogen and wear. For automotive manufacturers, optimising the metal-polymer friction pairs and reducing hydrogen-induced wear can improve the longevity and reliability of braking systems, gears, and other critical components. The strategies developed for mitigating hydrogen embrittlement and corrosion can be applied to various automotive materials, ensuring better performance in wet or high-stress environments. Beyond the automotive sector, industries such as aerospace, manufacturing equipment, and renewable energy could also benefit. In aerospace, more durable aircraft component materials would improve safety and performance. For renewable energy companies, particularly those involved in hydrogen fuel cell technology, the insights gained can enhance fuel cell components’ reliability, promoting sustainable energy solutions’ growth. The research provides valuable guidance for advancing material science and engineering in various industries, contributing to more efficient and reliable technologies.

## Conclusion

The comprehensive theoretical and experimental investigations to address hydrogen wear in brake friction pairs have yielded valuable insights into several crucial aspects. Firstly, an increase in surface-volume temperature, within the range equal to or surpassing that of the degrading polymer lining, corresponded to an elevation in the specific and equivalent electrical conductivity of the electrolyte concentration. This phenomenon is elucidated by the acceleration of ion movement facilitated by a reduction in medium viscosity and partial dehydration of ions, leading to a diminished hydrated ion radius and increased dissociation levels, particularly noticeable in weak electrolytes. Secondly, the composition selection for the components constituting the “metal-polymer” friction pair is a critical consideration. It is imperative to account for their interaction with water and hydrogen, ensuring that the potential of the electrode process for the metal in a neutral medium remains lower than that of water. Simultaneously, adherence to regulated intervals for the chemical composition ratio of the metal-polymer materials is emphasized, underscoring the importance of maintaining optimal performance and wear resistance. Lastly, a significant revelation involves establishing the dependence of the equivalent diffusion coefficient on the concentration of diffusing hydrogen atoms, particularly from metal surfaces such as pulleys or drum rims. This analysis is conducted at the thermal stabilization temperature, where the temperature gradient across the rim thickness is minimized within an equivalent thermal field. The research further delves into the concentration-dependent diffusion coefficient of hydrogen in iron, covering a broad range of concentrations, employing the quasi-chemical approximation. The study considers electronic and vibrational contributions to the chemical potential, providing a comprehensive understanding of the diffusion processes. Together, these findings significantly advance our comprehension of mechanisms to mitigate hydrogen wear in brake friction pairs. They offer valuable guidance for future material composition and design endeavours, promising enhanced durability and efficiency in braking systems.

## Data Availability

The datasets used and/or analysed during the current study available from the corresponding author on reasonable request.

## References

[1] Tian, J., Qi, X., Li, C. & Xian, G. Friction behaviors and wear mechanisms of multi-filler reinforced epoxy composites under dry and wet conditions: Effects of loads, sliding speeds, temperatures, water lubrication. *Tribol Int.* **179**, 108148. 10.1016/j.triboint.2022.108148 (2023).

[2] Bahadur, S. The development of transfer layers and their role in polymer tribology. *Wear* **245**, 92–99. 10.1016/S0043-1648(00)00469-5 (2000).

[3] Öchsner, R., Kluge, A., Zechel-Malonn, S., Gong, L. & Ryssel, H. Improvement of surface properties of polymers by ion implantation. *Nucl. Instruments Methods Phys. Res. Sect. B Beam Interact. Mater. Atoms* **80–81**, 1050–1054. 10.1016/0168-583X(93)90734-N (1993).

[4] Liao, C. et al. The cooperatively crosslinking between GO-COOH/TiO2 @PAO microcapsules and polyimide to improve the mechanical and tribological properties of PEEK/PI composites. *Tribol. Int.* **191**, 109209. 10.1016/j.triboint.2023.109209 (2024).

[5] Cheng, G., Chen, B., Guo, F., Xiang, C. & Jia, X. Research on the friction and wear mechanism of a polymer interface at low temperature based on molecular dynamics simulation. *Tribol Int.* **183**, 108396. 10.1016/j.triboint.2023.108396 (2023).

[6] Shrestha, K. et al. Intermediate nanofibrous charge trapping layer-based wearable triboelectric self-powered sensor for human activity recognition and user identification. *Nano Energy* **108**, 108180. 10.1016/j.nanoen.2023.108180 (2023).

[7] Zhang, L. et al. Preparation of phase change functional two-dimensional materials and the tribological properties. *Polym. Test.* **129**, 108278. 10.1016/j.polymertesting.2023.108278 (2023).

[8] Kumar, R. & Antonov, M. Self-lubricating materials for extreme temperature tribo-applications. *Mater. Today Proc.* **44**, 4583–4589. 10.1016/j.matpr.2020.10.824 (2021).

[9] Cao, Z., Xia, Y. & Chen, C. Fabrication of novel ionic liquids-doped polyaniline as lubricant additive for anti-corrosion and tribological properties. *Tribol Int.* **120**, 446–454. 10.1016/j.triboint.2018.01.009 (2018).

[10] Wu, Z. et al. Effect of 2D Cu-MOFs modified carbon spheres nanoparticles as an environmentally friendly lubricating additive on tribological properties. *Tribol Int.* **192**, 109321. 10.1016/j.triboint.2024.109321 (2024).

[11] Chen, Y., Li, D., Yang, W., Xiao, C. & Wei, M. Effects of different amine-functionalized graphene on the mechanical, thermal, and tribological properties of polyimide nanocomposites synthesized by in situ polymerization. *Polym. (Guildf)*. **140**, 56–72. 10.1016/j.polymer.2018.02.017 (2018).

[12] Lei, Y. et al. Proton irradiation-induced changes in the tribological performance of polyimide composites. *Tribol. Int.* **167**, 107427. 10.1016/j.triboint.2021.107427 (2022).

[13] Zou, K. et al. Poly(ionic liquid)s with amino acids counterions as multifunctional water-based additives contributing to green lubrication. *Tribol. Int.* **192**, 109295. 10.1016/j.triboint.2024.109295 (2024).

[14] Guo, H. et al. Tough, stretchable dual-network liquid metal-based hydrogel toward high-performance intelligent on-off electromagnetic interference shielding, human motion detection and self-powered application. *Nano Energy* **114**, 108678. 10.1016/j.nanoen.2023.108678 (2023).

[15] Liu, X. et al. Effect of SiC nanowires on adhesion and wear resistance of hydroxyapatite coating on AZ31 magnesium alloy. *J. Alloys Compd.* **960**, 170934. 10.1016/j.jallcom.2023.170934 (2023).

[16] Mohsenzadeh, R., Soudmand, B. H. & Shelesh-Nezhad, K. A combined experimental-numerical approach for life analysis and modeling of polymer-based ternary nanocomposite gears. *Tribol. Int.* **173**, 107654. 10.1016/j.triboint.2022.107654 (2022).

[17] Hovsepian, P. E. et al. Friction and wear behaviour of Mo–W doped carbon-based coating during boundary lubricated sliding. *Appl. Surf. Sci.* **366**, 260–274. 10.1016/j.apsusc.2016.01.007 (2016).

[18] Jaiswal, S. et al. Enhancing the lubricity and wear resistance of shape-memory-polymer via titanium carbide-based MAX and MXene. *Carbon N. Y.* **219**, 118790. 10.1016/j.carbon.2024.118790 (2024).

[19] Hirwani, J. K. et al. Epoxy (SU-8) polymer composites with ultra-high molecular weight polyethylene and hyaluronic acid fillers for hip prosthetic implant application. *Tribol. Int.* **167**, 107399. 10.1016/j.triboint.2021.107399 (2022).

[20] Varnava, C. K. & Patrickios, C. S. Polymer networks one hundred years after the macromolecular hypothesis: A tutorial review. *Polymer (Guildf)* **215**, 123322. 10.1016/j.polymer.2020.123322 (2021).

[21] Zhang, D. W., Yang, G. C., Lv, S. C., Tian, C. & Li, Z. J. Fretting behavior of static metal seal and testing apparatus for fretting friction with low/high temperature. *Tribol. Int.* **187**, 108676. 10.1016/j.triboint.2023.108676 (2023).

[22] Borawski, A. Impact of operating time on selected tribological properties of the friction material in the brake pads of passenger cars. *Materials (Basel)* **14**, 1–13. 10.3390/ma14040884 (2021). 10.3390/ma14040884 PMC7918745 33673339

[23] Han, J. H., Zhang, H., Chu, P. F., Imani, A. & Zhang, Z. Friction and wear of high electrical conductive carbon nanotube buckypaper/epoxy composites. *Compos. Sci. Technol.* **114**, 1–10. 10.1016/j.compscitech.2015.03.012 (2015).

[24] Ye, S. E. X. et al. Enhancing the tribological properties of boron nitride by bioinspired polydopamine modification. *Appl. Surf. Sci.* **529**, 147054. 10.1016/j.apsusc.2020.147054 (2020).

[25] Chen, C., Zhang, Z., Zhao, X. & Ye, L. Polyoxymethylene/graphene oxide-perfluoropolyether nano-composite with ultra-low friction coefficient fabricated by formation of superior interfacial tribofilm. *Compos. Part. Appl. Sci. Manuf.* **132**, 105856. 10.1016/j.compositesa.2020.105856 (2020).

[26] Najafi Hajivar, I. & Kokabi, M. Polymer-network hydrogel facilitated synthesis of Ca-α-SiAlON balls composed of nanoparticles. *Ceram. Int.* **39**, 3321–3327. 10.1016/j.ceramint.2012.10.021 (2013).

[27] Kumar, A., Kumar, M. & Tailor, S. Self-lubricating composite coatings: A review of deposition techniques and material advancement. *Mater. Today Proc.*10.1016/j.matpr.2023.01.035 (2023).35966411

[28] Ren, S. L., Yang, S. R. & Zhao, Y. P. Derivatization, characterization, and tribological behavior of an amine-terminated polymer surface. *Appl. Surf. Sci.* **227**, 293–299. 10.1016/j.apsusc.2003.12.004 (2004).

[29] Yu, P. et al. Significance of g-C3N4 nanosheets for enhancing tribological performance of epoxy subjected to starved lubrication. *Tribol. Int.* **174**, 107762. 10.1016/j.triboint.2022.107762 (2022).

[30] Higham, P. A., Stott, F. H. & Bethune, B. The influence of polymer composition on the wear of the metal surface during fretting of steel on polymer. *Wear* **47**, 71–80. 10.1016/0043-1648(78)90204-1 (1978).

[31] Zhu, Y. et al. Effect of modified nano boron nitride on tribological performance of resin-based friction material paired with copper dual disk. *Tribol. Int.* **168**, 107429. 10.1016/j.triboint.2022.107429 (2022).

[32] Kharitonov, A. P. Direct fluorination of polymers—from fundamental research to industrial applications. *Prog. Org. Coat.* **61**, 192–204. 10.1016/j.porgcoat.2007.09.027 (2008).

[33] Makowski, S., Schaller, F., Weihnacht, V., Englberger, G. & Becker, M. Tribochemical induced wear and ultra-low friction of superhard ta-C coatings. *Wear* 392–393. 10.1016/j.wear.2017.08.015 (2017).

[34] Mukhametishina, R. M. Hydrogen wear of parts of road-building machines. *Izv. KGSAU* **4**, 334–338 (2015).

[35] Garkunov, D. N. Tribotechnics, hydrogen wear of machine parts. In *Proc. USTU* (eds Garkunov, D.N., Suranov, G.I. & Khrustalev, Y.A.) 260 (2007).

[36] Garkunov, D. N. On the method of increasing the durability of wheel and brake pairs. *Eff. Wearlessness Tribotechnol.* **1**, 32–36 (1998).

[37] Galaktionova, N. A. Hydrogen in metals. *Iz-vo Metallurgy* 303 (1967).

[38] Kindrachuk, M. V., Volchenko, D. A. & Volchenko, N. A. Influence of a water conduit on the wear resistance of materials in friction pairs of brake devices. *Phiz -Khim Mech. Mater.* **53**, 135–141 (2017).

[39] Kernytskyy, I. et al. Complex heat exchange in friction steam of brakes. *Energies* **15**. 10.3390/en15197412 (2022).

[40] Shalygin, M. G. *Wear of the subroughness of friction surfaces in a hydrogen-containing medi-um* (2017).

[41] Yudin, V. M. Tribochemistry of hydrogen wear. MGUPS. 282 (2004).

[42] Liu, D. et al. Effect of tempering temperature and carbide free bainite on the mechanical characteristics of a high strength low alloy steel. *Mater. Sci. Eng. A*. **371**, 40–44. 10.1016/s0921-5093(03)00270-3 (2004).

[43] Smirnov, L. I. Diffusion and behavioral patterns of the hydrogen subsystem in metal-hydrogen systems. *Author Diss. Doc. Tech. Sci.* **36** (2003).

[44] Baltes, L., Patachia, S., Tierean, M., Ekincioglu, O. & Ozkul, H. M. Photoactive glazed polymer-cement composite. *Appl. Surf. Sci.* **438**, 84–95. 10.1016/j.apsusc.2017.09.068 (2018).

[45] Mohsenzadeh, R., Soudmand, B. H. & Shelesh-Nezhad, K. Load-bearing analysis of polymer nanocomposite gears using a temperature-based step loading technique: Experimental and numerical study. *Wear* 514–515. 10.1016/j.wear.2022.204595 (2023).

[46] Sharma, V., Timmons, R. B., Erdemir, A. & Aswath, P. B. Interaction of plasma functionalized TiO2 nanoparticles and ZDDP on friction and wear under boundary lubrication. *Appl. Surf. Sci.* **489**, 372–383. 10.1016/j.apsusc.2019.05.359 (2019).

[47] Zhang, H. et al. Preparation, mechanical and anti-friction performance of MXene/polymer composites. *Mater. Des.* **92**, 682–689. 10.1016/j.matdes.2015.12.084 (2016).

[48] Cui, L. et al. Metal ion reinforced hydrogel/Ti6Al4V bionic composite joint bearing interface with extraordinary mechanical and biotribological properties. *Tribol. Int.* **189**, 109024. 10.1016/j.triboint.2023.109024 (2023).

[49] Yu, H. et al. Transformation mechanism between the frictional interface under dioctyl sebacate lubrication. *Tribol Int.* **155**, 106745. 10.1016/j.triboint.2020.106745 (2021).

[50] Yan, S., Xin, Z., Xue, Y. & Zhang, H. Improved lubrication and wear resistance of gallium-matrix liquid metal containing molybdenum diselenide nanoparticles under heavy load conditions. *Wear* 528–529. 10.1016/j.wear.2023.204987 (2023).

[51] Guo, J. et al. Surface-modified Ti3C2Tx MXene as anti-wear and extreme pressure additive for PFPE supramolecular gel. *Tribol. Int.* **186**, 108611. 10.1016/j.triboint.2023.108611 (2023).

[52] Wang, B. et al. Zwitterionic polymer brush-functionalized dual-crosslinked hydrogel via subsurface-initiated atom transfer radical polymerization for anti-fouling applications. *Prog. Org. Coat.* **183**, 107729. 10.1016/j.porgcoat.2023.107729 (2023).

[53] Ma, X. et al. Flexural strength and wear resistance of C/C–SiC brake materials improved by introducing SiC ceramics into carbon fiber bundles. *Ceram. Int.* **47**, 24130–24138. 10.1016/j.ceramint.2021.05.124 (2021).

[54] Dzhanakhmedov, A. K. & Volchenko, N. A. Tribology: Friction, wear and lubrication. *Apostrophe-A* **640** (2019).

[55] Myshkin, N. K. & Konchits, V. V. Friction and wear of metal-composite electrical contacts. *Wear* **158**, 119–140. 10.1016/0043-1648(92)90034-6 (1992).

[56] Salem, A., Guezmil, M., Bensalah, W. & Mezlini, S. Tribocorrosion behavior of 316 L and HDPE composites for orthopedic application. *Mater. Today Commun.* **31**, 103582. 10.1016/j.mtcomm.2022.103582 (2022).

[57] Duan, L. et al. Tribological performance and anti-wear mechanism of cadmium-based phosphate microspheres as lubricant additives. *Wear* 534–535. 10.1016/j.wear.2023.205151 (2023).

[58] Cyriac, F., Yi, T. X., Poornachary, S. K. & Chow, P. S. Effect of temperature on tribological performance of organic friction modifier and anti-wear additive: Insights from friction, surface (ToF-SIMS and EDX) and wear analysis. *Tribol. Int.* **157**, 106896. 10.1016/j.triboint.2021.106896 (2021).

[59] Wu, L. et al. Facile synthesis of CuO/g-C3N4 hybrids for enhancing the wear resistance of polyimide composite. *Eur. Polym. J.* **116**, 463–470. 10.1016/j.eurpolymj.2019.04.041 (2019).

[60] Shan, Z. et al. Cellulose-armored CNTs-soft metal hybrid nanomaterials to improving the friction-reduction and anti-wear performance of bio-lubricants. *Tribol. Int.* **191**, 109085. 10.1016/j.triboint.2023.109085 (2024).

[61] Wang, H. et al. Tribological properties of graphene oxide reinforced PPTA/PTFE composites. *J. Mater. Res. Technol.* **23**, 3505–3514. 10.1016/j.jmrt.2023.02.032 (2023).

[62] Opia, A. C. et al. Improving tribological properties and shear stability of base lubricant using Eichhornia crassipes carboxylmethyl cellulose polymer under different conditions. *Ind. Crops Prod.* **180**, 114741. 10.1016/j.indcrop.2022.114741 (2022).

[63] Wu, Y. et al. Static friction measurement methodology for the assessment of performance of industrial valves at high temperatures: Case study for a nickel-based alloy coating. *Tribol Int.* **192**, 109237. 10.1016/j.triboint.2023.109237 (2024).

[64] Huang, Y. et al. Photothermally responsive and durable polydopamine-modified MXene-PNIPAM hydrogels for smart friction regulation. *Tribol. Int.* 109435. 10.1016/j.triboint.2024.109435 (2024).

[65] Pöllinger, A. et al. Thermo-mechanical properties and internal architecture of PI composites for high-pressure hydrogen applications. *Polymer (Guildf)* **289**, 126500. 10.1016/j.polymer.2023.126500 (2023).

[66] Alefeld, G. & Volkl, I. (eds). Hydrogen in metals. In *Hydrog. Met.* I–475, II–430 (1981).

[67] Volchenko, N. A. Electrochemistry with double electrical layers in frictional interaction metal-polymer tribolink. In *IOP Conf. Ser. Mater. Sci. Eng.* (eds Volchenko, N. A., Krasin, P. S., Volchenko, D. A., Voznyi, A. V.) 032059 (2018).

[68] Volchenko, N. A. Pulse-contact frictional interaction of micropro-trussions of friction pairs of brake devices. In (eds Volchenko, D. A., Volchenko, N. A., Polyakov, P. A., Fedotov, E. S. & Evchenko, A. S.) *IOP Conf. Ser. Mater. Sci. Eng.*) 012194 (2019).

[69] Dzhanakhmedov, A. K., Volchenko, D. A. & Volchenko, N. A. Design and verification calculation of friction units of band-shoe brakes of drilling winches: Standard, Baku Apostr. (n.d.) 311.

[70] Rodríguez, H. & Rogers, R. D. Liquid mixtures of ionic liquids and polymers as solvent systems. *Fluid Phase Equilib.* **294**, 7–14. 10.1016/j.fluid.2009.12.036 (2010).

[71] Cui, Y. et al. Fabrication of MXene@Fe3O4@PNA composite with photothermal effect as water-based lubricant additive. *Chem. Eng. J.* **469**, 143880. 10.1016/j.cej.2023.143880 (2023).

[72] Lyubarsky, I. M. & Palatnik, L. S. Metallophysics of friction. *Metallurgy* **1976** (1976).

[73] Kostetsky, B. I., Bosovsky, I. G. & Karaulov, A. K. Surface strength of materials during friction. *Technique* **292** (1976).

[74] Terentiev, V. F. Tribotechnical materials science. *Krasnoyarsk* (2000).

[75] Becerra-Mora, N., Rajasekaran, P. R., Voss, K. O., Kollipara, V. K. & Kohli, P. Device fabrication on curvilinear two-dimensional surfaces using polymer probes. * Polymer (Guildf)* **218**, 123521. 10.1016/j.polymer.2021.123521 (2021).

[76] Zhang, B. et al. Sliding friction and wear behaviors of surface-coated natural serpentine mineral powders as lubricant additive. *Appl. Surf. Sci.* **257**, 2540–2549. 10.1016/j.apsusc.2010.10.019 (2011).

[77] Wan, C. et al. Synergism lubrication of graphene and carbon nanotube in polymeric composites under drying sliding condition. *Appl. Surf. Sci.* **630**, 157430. 10.1016/j.apsusc.2023.157430 (2023).

[78] He, J. et al. Tribological properties of physically modified fluorinated graphene and soluble starch hybrid as water-based lubricating additive system. *Tribol. Int.* **183**, 108412. 10.1016/j.triboint.2023.108412 (2023).

[79] Wang, F. et al. A Polytetrafluoroethylene@Polyacrylonitrile core-shell composite with high tribological performance. *Polymer (Guildf)* **289**, 126493. 10.1016/j.polymer.2023.126493 (2023).

[80] Sánchez-Rodríguez, C. et al. Ionic liquid lubricants of PLA. New self-lubricating (PLA + ionic liquid) materials,.*Tribol. Int.* **186**, 108630. 10.1016/j.triboint.2023.108630 (2023).

[81] Chen, H. et al. High performance composite films assembled on metal substrates with graphene oxide. *Surf. Coat. Technol.* **441**, 128527. 10.1016/j.surfcoat.2022.128527 (2022).

[82] Wojciechowski, Ł. et al. Towards the superlubricity of polymer–steel interfaces with ionic liquids and carbon nanotubes. *Tribol. Int.* **191**, 109203. 10.1016/j.triboint.2023.109203 (2024).

[83] Zhou, S. et al. A review on tribology, characterization and lubricants for water-based drilling fluids. *Geoenergy Sci. Eng.* **229**, 212074. 10.1016/j.geoen.2023.212074 (2023).

[84] Maleki, F., Razmi, H., Rashidi, M. R., Yousefi, M. & Ghorbani, M. Recent advances in developing electrochemical (bio)sensing assays by applying natural polymer-based electrospun nanofibers: A comprehensive review. *Microchem J.* **197**, 109799. 10.1016/j.microc.2023.109799 (2024).

[85] Kerni, L., Raina, A. & Haq, M. I. U. Friction and wear performance of olive oil containing nanoparticles in boundary and mixed lubrication regimes. *Wear* 426–427. 10.1016/j.wear.2019.01.022 (2019).

[86] Puhan, D., Jiang, S. & Wong, J. S. S. Effect of carbon fiber inclusions on polymeric transfer film formation on steel. *Compos. Sci. Technol.* **217**, 109084. 10.1016/j.compscitech.2021.109084 (2022).

[87] Trajkovski, A. et al. Glycerol aqueous solutions for the enhanced tribological behaviour of polymer composites sliding against steel. *Tribol. Int.* **192**, 109173. 10.1016/j.triboint.2023.109173 (2024).

[88] Sharifahmadian, O., Pakseresht, A., Amirtharaj Mosas, K. K. & Galusek, D. Doping effects on the tribological performance of diamond-like carbon coatings: A review. *J. Mater. Res. Technol.* **27**, 7748–7765. 10.1016/j.jmrt.2023.11.132 (2023).

[89] Baskov, V., Ignatov, A. & Polotnyanschikov, V. Assessing the influence of operating factors on the properties of engine oil and the environmental safety of internal combustion engine. *Transp. Res. Procedia*. **50**, 37–43. 10.1016/j.trpro.2020.10.005 (2020).

[90] Fan, X. et al. MoS2 functionalized lithium complex soap with enhanced thickening net structure toward high-performance thickener. *Chem. Eng. J.* **478**, 147445. 10.1016/j.cej.2023.147445 (2023).

[91] Trajkovski, A. et al. Glycerol aqueous solutions for the enhanced tribological behaviour of polymer composites sliding against steel. *Tribol Int.* **192**, 109173. 10.1016/j.triboint.2023.109173 (2024).

[92] Sánchez-Rodríguez, C. et al. Ionic liquid lubricants of PLA. New self-lubricating (PLA + ionic liquid) materials. *Tribol. Int.* **186**, 0–11. 10.1016/j.triboint.2023.108630 (2023).

[93] Krishnadoss, V. et al. Programmable bio-ionic liquid functionalized hydrogels for in situ 3D bioprinting of electronics at the tissue interface. *Mater. Today Adv.* **17**, 100352. 10.1016/j.mtadv.2023.100352 (2023).

[94] Usui, T. et al. Mechanical and frictional properties of aesthetic orthodontic wires obtained by hard chrome carbide plating. *J. Dent. Sci.* **13**, 151–159. 10.1016/j.jds.2017.07.003 (2018). 30895111 10.1016/j.jds.2017.07.003 PMC6388802

[95] Zhang, L. et al. Macro-superlubric triboelectric nanogenerator based on tribovoltaic effect. *Matter* **5**, 1532–1546. 10.1016/j.matt.2022.02.021 (2022).

[96] Li, Y., Nie, D. & Cai, Z. The performance analysis of screw pump stator elastomers: Polyamide 6/hydrogenated nitrile blends—Mechanical, oil resistance and tribological properties. *Polym. Test.* **128**, 108226. 10.1016/j.polymertesting.2023.108226 (2023).

[97] Le, T. H. et al. Surfactant-free GO-PLA nanocomposite with honeycomb patterned surface for high power antagonistic bio-triboelectric nanogenerator. *J. Sci. Adv. Mater. Devices*. **7**, 100392. 10.1016/j.jsamd.2021.08.005 (2022).

[98] González-Hernández, A., Morales-Cepeda, A. B., Caicedo, J. C., Amaya, C. & Olive-Méndez, S. F. Structure, functional groups analysis and tribo-mechanical behavior of carbide and nitride coatings deposited on AISI 1060 substrates by RF-magnetron sputtering. *J. Mater. Res. Technol.* **18**, 5432–5443. 10.1016/j.jmrt.2022.04.075 (2022).

[99] Shi, X. et al. Advanced strategies for marine antifouling based on nanomaterial-enhanced functional PDMS coatings. *Nano Mater. Sci.*10.1016/j.nanoms.2023.12.005 (2024).

[100] Chen, K. Y. et al. Recent progress in graphene-based wearable piezoresistive sensors: From 1D to 3D device geometries. *Nano Mater. Sci.* **5**, 247–264. 10.1016/j.nanoms.2021.11.003 (2023).

[101] Kania, D. et al. Lubricity performance of non-ionic surfactants in high-solid drilling fluids: A perspective from quantum chemical calculations and filtration properties. *J. Pet. Sci. Eng.* **207**, 109162. 10.1016/j.petrol.2021.109162 (2021).

[102] Collins, I. R. et al. Transition from oil & gas drilling fluids to geothermal drilling fluids. *Geoenergy Sci. Eng.* **233**, 212543. 10.1016/j.geoen.2023.212543 (2024).

[103] Zheng, Q. J. et al. Interparticle forces and their effects in particulate systems. *Powder Technol.* **436**. 10.1016/j.powtec.2024.119445 (2024).

[104] Rosa-Sainz, A., Centeno, G., Silva, M. B. & Vallellano, C. Experimental investigation of polycarbonate sheets deformed by SPIF: Uormability, micro-mechanisms of failure and temperature analysis. *J. Mater. Res. Technol.* **25**, 7546–7565. 10.1016/j.jmrt.2023.07.075 (2023).

